# Atomistic mechanisms of hard carbon formation from polyvinylidene chloride

**DOI:** 10.1039/d6sc05974k

**Published:** 2026-10-08

**Authors:** Litong Wu, Zitong Wu, Zakariya El-Machachi, Volker L. Deringer

**Affiliations:** a Inorganic Chemistry Laboratory, Department of Chemistry, University of Oxford Oxford UK volker.deringer@chem.ox.ac.uk

## Abstract

Hard carbons are a class of disordered materials with widespread application in energy storage. Despite decades of research, their atomistic formation mechanisms have remained elusive, due to the difficulty of both *in situ* experimental characterization and first-principles simulations. Here, we describe the formation mechanism of hard carbon from the thermal decomposition of polyvinylidene chloride (PVDC), using first-principles-quality simulations with a bespoke machine-learned interatomic potential model. Our results indicate a two-stage process, consisting of (i) radical-mediated dehydrochlorination, which generates reactive unsaturated carbon sites, and (ii) progressive carbon–carbon cross-linking followed by thermally activated rearrangement into an extended sp^2^-bonded network. We provide an atomistic account of non-hexagonal ring motifs emerging during pyrolysis, supporting the established theory that these motifs induce the intrinsic curvature that frustrates graphitic ordering in hard carbons.

## Abstract 2

Machine-learning-driven simulations reveal how polyvinylidene chloride (PVDC) decomposes and transforms into hard carbon at the atomic scale.

## Graphical abstract

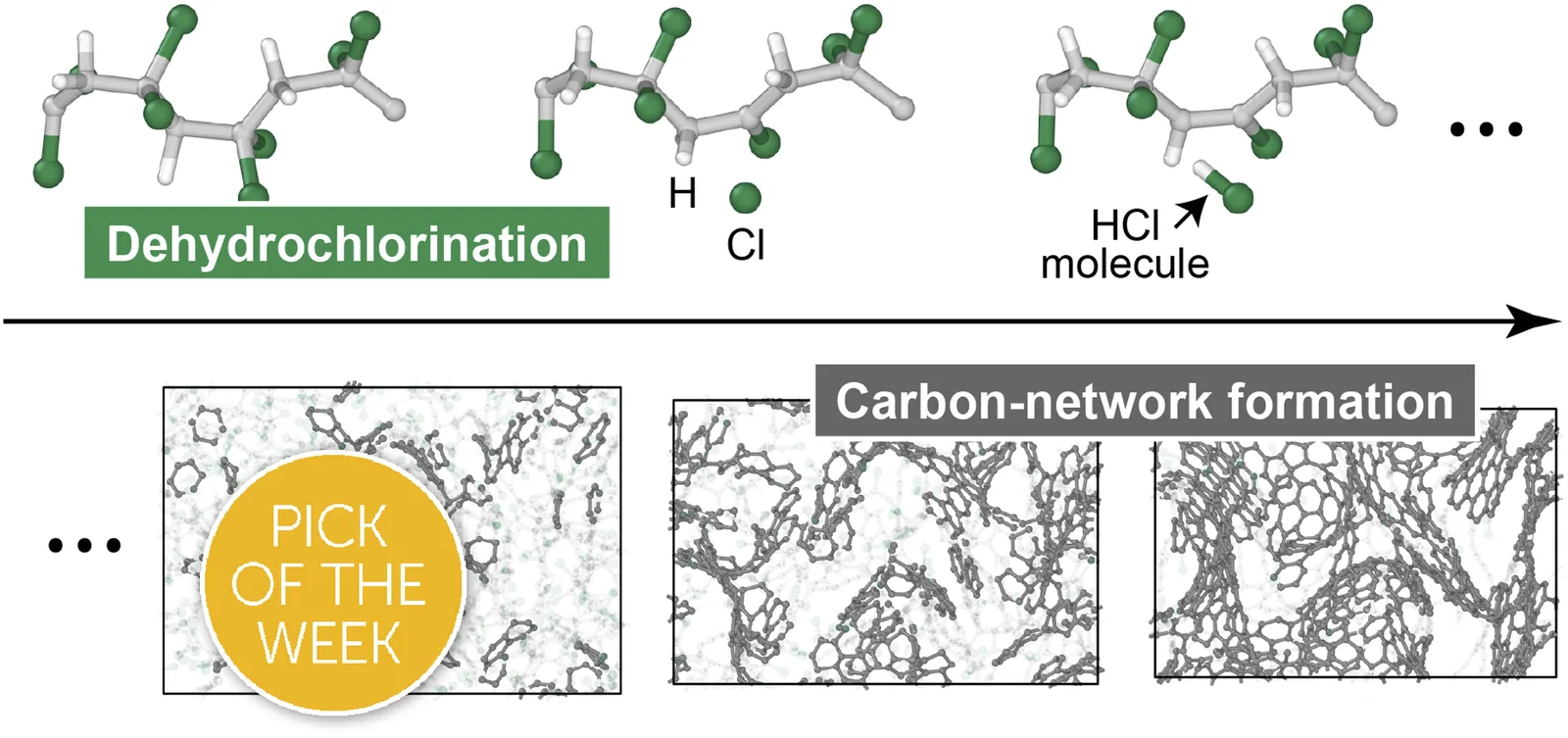

## Introduction

In a multi-component device as intricate as a battery, achieving electrochemical compatibility across all components is crucial. The discovery of viable anode hosts has been a key challenge in this regard, as borne out by the historical development of both lithium-ion batteries (LIBs) and sodium-ion batteries (SIBs).^1–3^ The first commercial LIB, introduced in 1991, employed petroleum-coke-derived soft carbon;^4^ subsequent work explored hard carbon synthesized from polyfurfuryl alcohol resin to achieve higher reversible capacity.^5^ Graphite later became the dominant LIB anode upon the recognition that its lithiation could be stabilized in ethylene-carbonate-based electrolytes through formation of a solid-electrolyte interphase.^6–8^ However, the situation is different in SIBs: graphite does not form thermodynamically favourable Na intercalation compounds under typical battery operating conditions, whereas hard carbons readily accommodate Na ions within their disordered structure.^9–11^ Since the first report of reversible Na-ion storage in glucose-derived hard carbon in 2000,^12^ hard carbons have become the leading anode material for contemporary SIB technologies.^1,13^

The recognition of hard carbons as a distinct class of disordered carbonaceous materials, structurally different from both crystalline graphite and fully amorphous carbon, can be traced back to early X-ray diffraction (XRD) studies in the 1940s.^14,15^ The subsequent systematic distinction between hard and soft carbons (then termed non-graphitizing and graphitizing carbons, respectively) was established through the foundational studies of Franklin.^16–18^ Hard carbons are disordered materials that cannot be transformed into crystalline graphite even at temperatures as high as 3000 °C, whereas soft carbons are disordered carbons that can.^19^ These materials are typically synthesized by heating polymer precursors to approximately 1000 °C in the absence of oxygen (“pyrolysis”),^20^ with the choice of precursor playing a central role in determining the nature of the resulting carbon.^15,21^ A classic example is a pair of chlorinated polymers, polyvinylidene chloride (PVDC) and polyvinyl chloride (PVC), whose pyrolysis yields hard and soft carbons, respectively.^18,22^ As a first step towards understanding this contrast, we will here use PVDC as a model system to investigate how precursor structure directs hard-carbon formation.^23–27^

Over the decades, experiments have provided key insights into the structure and formation of PVDC-derived hard carbons. Thermogravimetric analysis and mass spectrometry revealed a two-stage mass loss during PVDC pyrolysis: HCl evolution at around 250 °C, and a minor release of hydrocarbon species near 500 °C.^27,28^ Infrared spectroscopy indicated that early carbonization involved the loss of C–Cl functionalities, followed by the development of conjugated C=C features.^23^ Electron spin resonance measurements showed paramagnetic signals during initial dehydrochlorination; this signal then diminished and semiconducting behaviour emerged due to electron delocalization over the conjugated system that developed.^29,30^ XRD studies confirmed that, although structural ordering increased with pyrolysis temperature, the crystallite dimensions along the *a*- and *c*-axes remained limited (*L*_*a*_, *L*_*c*_ < 5 nm) even at 3000 °C. The persistence of a broad (002) reflection indicated a large and stable inter-layer spacing, in contrast to the layer contraction expected to be observed during graphitization.^18,22,24,27^

The challenge, however, lies in translating these experimental signatures into a coherent atomistic understanding. Franklin attributed hard carbons' resistance to graphitization to randomly oriented graphitic domains, interconnected by covalent cross-links that impede rearrangement into a parallel structure, although the nature of these cross-links remains unclear.^18^ In the 1980s, Oberlin described the structural evolution in terms of forming and aligning basic structural units into locally oriented domains, with precursor-dependent viscosity governing domain growth and graphitizability.^31,32^ Following the discovery of fullerenes, Harris and Tsang proposed a model in which non-hexagonal rings introduce curvature into defective graphene-like fragments.^26,33,34^ This view was later supported by aberration-corrected transmission electron microscopy studies by Guo *et al.* (ref. 35) and Allen *et al.* (ref. 36). Recently, Fogg *et al.* showed that PVDC-derived hard carbon can be transformed into highly crystalline graphite by repeated rapid heating pulses, proposing that selective removal of non-hexagonal defects enables graphitization.^37^

While these models provide a structural framework for hard carbons, they do not explain how the proposed topological motifs arise from the reactive chemistry of a polymer precursor. Experimentally, chemical reactions are probed mainly through indirect and ensemble-averaged signatures. Computationally, resolving the reactive sequence requires an atomistic description of bond-breaking and bond-forming events across length and time scales beyond the reach of conventional first-principles methods. Reactive molecular dynamics (MD) simulations using empirical force fields, particularly ReaxFF,^38^ have provided valuable insights into the pyrolysis-driven carbonization of various polymer systems.^39–42^ However, the accuracy and transferability of such empirical force fields remain constrained by their parameterization, particularly for chemically complex high-temperature pathways involving halogenated precursors. Machine-learned interatomic potentials (MLIPs) offer a practical route forward by combining near first-principles fidelity with computational efficiency,^43–45^ both of which are important for describing the high-temperature structural evolution in chemically complex carbonaceous materials. Recent polymer-focused datasets and foundation-model efforts further highlight the growing role of machine-learning approaches in atomistic polymer modelling.^46,47^

Herein, we provide an atomistic account of the chemical and structural transformations underlying PVDC pyrolysis. We first characterize the radical-mediated dehydrochlorination cascade and examine how its regioselectivity evolves as the carbon network matures. We then identify a stepwise carbon cross-linking mechanism driven by the emergence of unsaturated carbon sites. Finally, we show how the initially defect-rich carbon cluster rearranges into a curved sp^2^ network, in which the persistence of odd-membered rings frustrates planar graphitic ordering. Using a bespoke graph-based MLIP model together with a specialized MD protocol, our simulation connects precursor-level reaction chemistry to the emergence of topological disorder, providing an atomistic perspective on the non-graphitizing character of PVDC-derived hard carbon.

## Results and discussion

### Machine-learning-driven simulation of PVDC pyrolysis

We performed MD simulations of PVDC pyrolysis with a bespoke MLIP fitted using the MACE architecture,^48^ starting from a supercell constructed from the single-crystal XRD-refined structure.^49^ Details of the dataset and model-training procedure are described in the Methods section, while numerical validation and robustness tests, including targeted validation relevant to the reactive processes, are presented in Section S1.2 of the SI. Representative snapshots of the system throughout the pyrolysis process, viewed along the polymer-chain direction, are shown in Fig. 1a. These snapshots provide an atomistic view of the transformation: as the temperature increases, dehydrochlorination generates unsaturated carbon sites that subsequently cross-link and progressively coalesce into a disordered hard-carbon network.

**Fig. 1:**
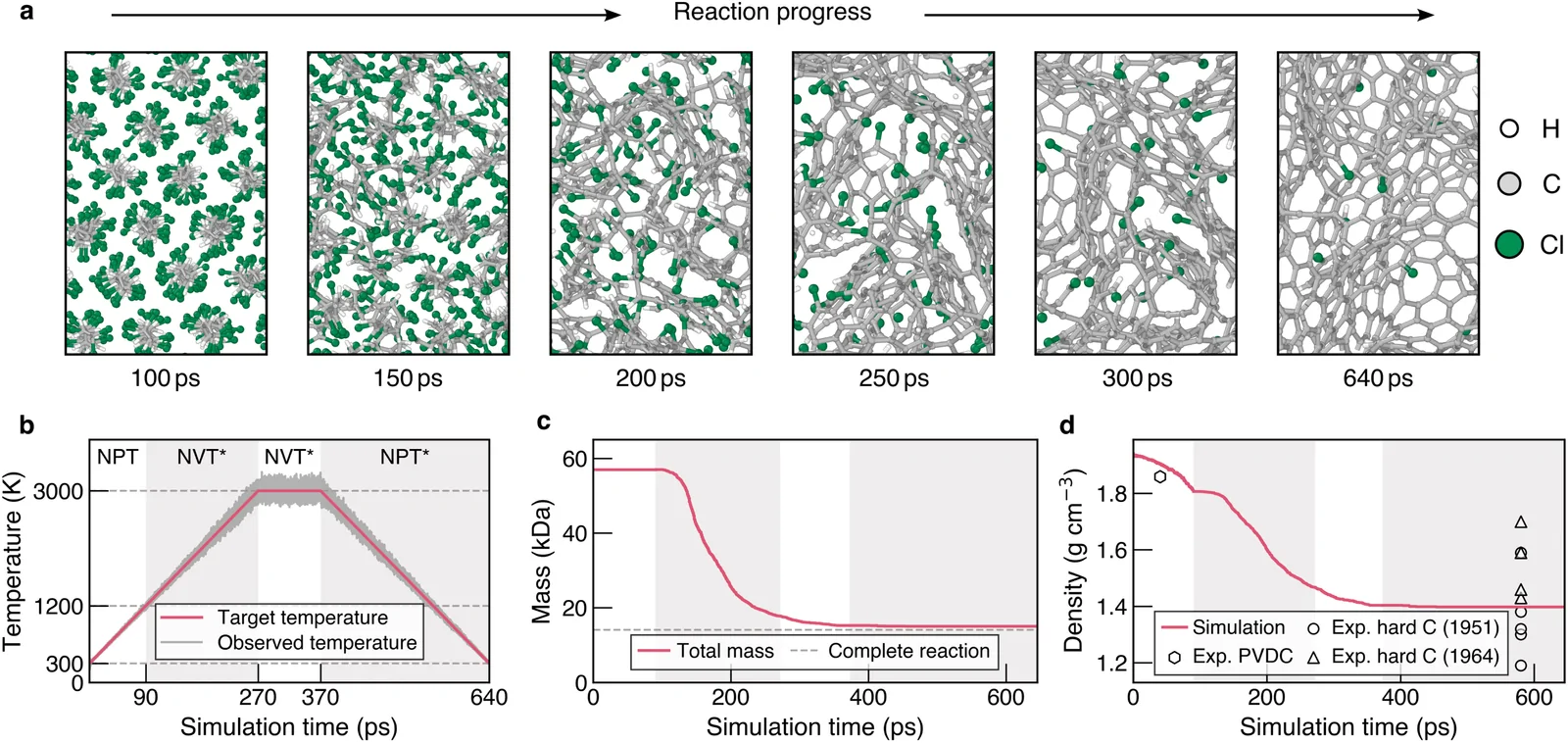
Machine-learning-driven simulation of PVDC pyrolysis. (a) Representative snapshots from a 640 ps MD trajectory using a bespoke MLIP model. The initial snapshot shows PVDC viewed along the polymer-chain direction. The reaction proceeds *via* elimination of chlorine and hydrogen atoms as HCl molecules, followed by cross-linking of the resulting unsaturated carbon chains into a curved sp^2^-rich network. (b) Schematic overview of the MD protocol. The system was heated from 300 K to 3000 K at a rate of 10^13^ K s^−1^, annealed at 3000 K for 100 ps, and quenched at the same rate. Ensembles used for each stage are indicated at the top; asterisks denote fixed-interval HCl removal combined with cell rescaling. The instantaneous temperature during the simulation is tracked in grey. (c) Mass change associated with simulated HCl removal. The grey dashed line marks the mass expected after complete HCl elimination. (d) Evolution of the system density. Experimental densities of commercial PVDC (hexagon^49^) and PVDC-derived hard carbons (circles^18^ and triangles^50^) are shown for comparison. Background shading is used to highlight the different simulation stages defined in panel (b), and is used in the same way in Fig. 2a and 3a.

Fig. 1b illustrates the MD protocol, with the instantaneous simulation temperature superimposed on the target temperature profile. The system was heated from 300 to 3000 K at a rate of 10^13^ K s^−1^, with a switch from the NPT to the NVT* ensemble at 1200 K to avoid simulation-cell instability caused by the evolution of gaseous molecules at higher temperature. We use asterisks to indicate simulations in which HCl molecules were periodically removed and the simulation cell isotropically rescaled to maintain a physically realistic density (see Methods). The system was then annealed at 3000 K for 100 ps and quenched back to 300 K in the NPT* ensemble, again at a rate of 10^13^ K s^−1^. The four stages of the MD protocol are indicated by alternating background shading in our plots.

Given that PVDC consists of alternating hydrogen and chlorine substituents along the polymer backbone, its primary thermal decomposition pathway is dehydrochlorination:
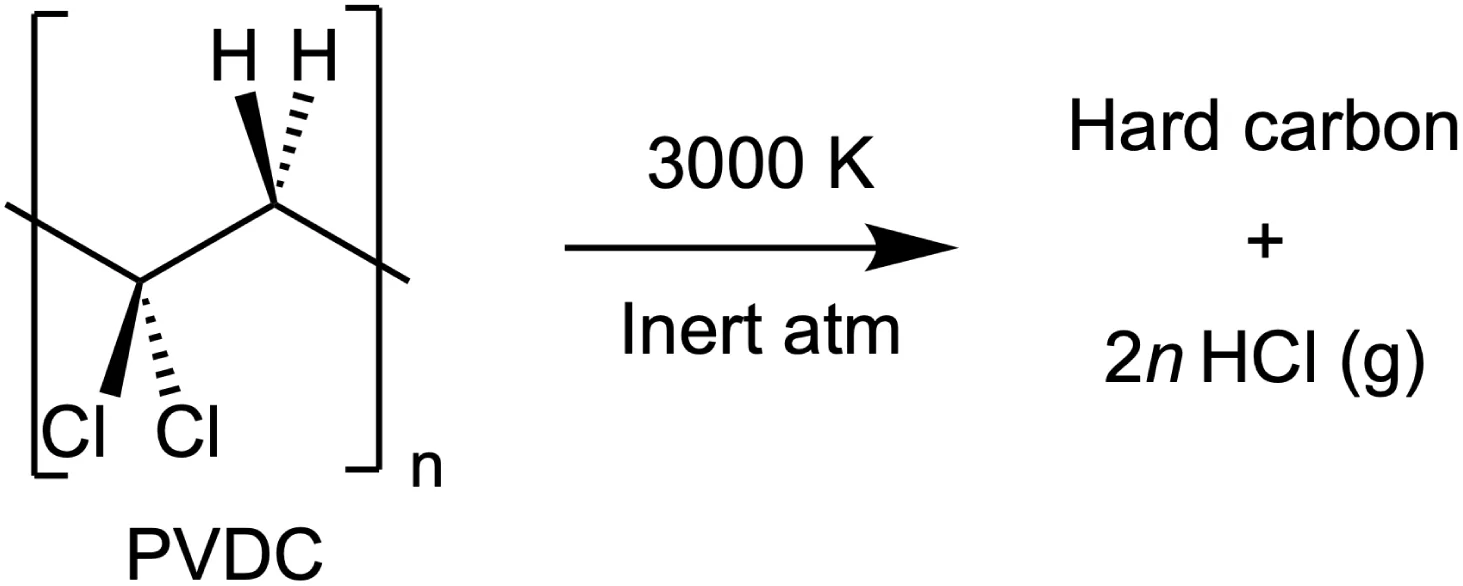


This behaviour is well captured by our simulation: HCl accounts for 83.1% of all molecular products detected and is the only product removed (SI, Section S2.1). Although transient H_2_ and Cl_2_ molecules are occasionally formed, their low abundance is consistent with experimental studies identifying HCl as the principal volatile product of PVDC degradation.^27,28,51,52^ An HCl-only removal protocol was therefore adopted to target the experimentally dominant volatile-loss pathway.

The resulting mass evolution due to the removal of HCl molecules formed is plotted in Fig. 1c. The first HCl removal occurs after 100 ps, when the simulation temperature reaches approximately 1300 K. Since all HCl molecules produced within each 1-ps interval are removed at the end of that interval during the NVT* stages, the resulting mass profile provides a measure of the reaction kinetics. Dehydrochlorination appears to be consistent with pseudo-first-order kinetics under the imposed heating protocol, where the reaction rate decreases as the available H- and Cl-containing sites are consumed (SI, Section S2.2). The last HCl removal occurred during quenching, when the system reached 2200 K, by which point 97.9% of the total HCl had been eliminated.

To assess the physical validity of the MD protocol, the density evolution is presented in Fig. 1d. The initial density of the simulation cell is slightly higher than that typically reported for bulk PVDC,^49^ because the starting structure is derived from a defect-free single-crystal XRD model. During the NPT heating phase, the density decreased slightly due to thermal expansion. Throughout the NVT* stages, the density is governed by the rescaling parameters and remains constant over the first 10 ps, as no HCl has yet been eliminated. A decrease in density began at 100 ps, concomitant with the onset of dehydrochlorination, and subsequently proceeded at a rate that mirrors the reaction rate. At the start of the quenching stage, despite switching to an NPT* ensemble at 3000 K, the density remained approximately constant at 1.4 g cm^−3^. This behaviour indicates that the carbon network has already developed substantial mechanical rigidity, consistent with earlier experimental evidence that dehydrochlorination-generated polyene chains undergo early inter-chain cross-linking which effectively “freezes” the structure.^51^ As the density evolution under this simulation protocol is sensitive to the chosen rescaling parameter, the parameter was calibrated such that the final density of the simulated hard carbon falls within the reported range for PVDC-derived hard carbons.^18,50^

Beyond density, the final carbon structure was further compared with experimental PVDC-derived hard carbon using radial distribution functions, simulated X-ray diffraction, and real-space structural descriptors (SI, Section S1.4). The comparison shows good agreement in local C–C structure and interlayer separation, while the reduced long-range ordering is likely a result of the limited dimensions of the simulation cell.

### Dehydrochlorination mechanism

Having characterized the macroscopic evolution of the system during pyrolysis, we next analyse the bond-level changes underlying dehydrochlorination. An examination of the bond-count evolution in Fig. 2a provides insight into the mechanism. The overall decrease in C–Cl and C–H bond counts is consistent with the observed mass loss due to HCl removal, while the increase in C–C bonds at around 150 ps suggests the onset of carbon cross-linking. However, the expanded view in the inset of Fig. 2a reveals that the cleavage of C–Cl bonds precedes that of C–H bonds; the time at which 1% of all C–Cl bonds had remained dissociated for longer than 0.2 ps preceded that for C–H bonds by 42.4 ps. In addition, the C–C bond count undergoes a shallow decline by 4.4% at 146.4 ps before increasing again, implying thermally induced or radical-driven C–C scission prior to net carbon-network growth.

**Fig. 2:**
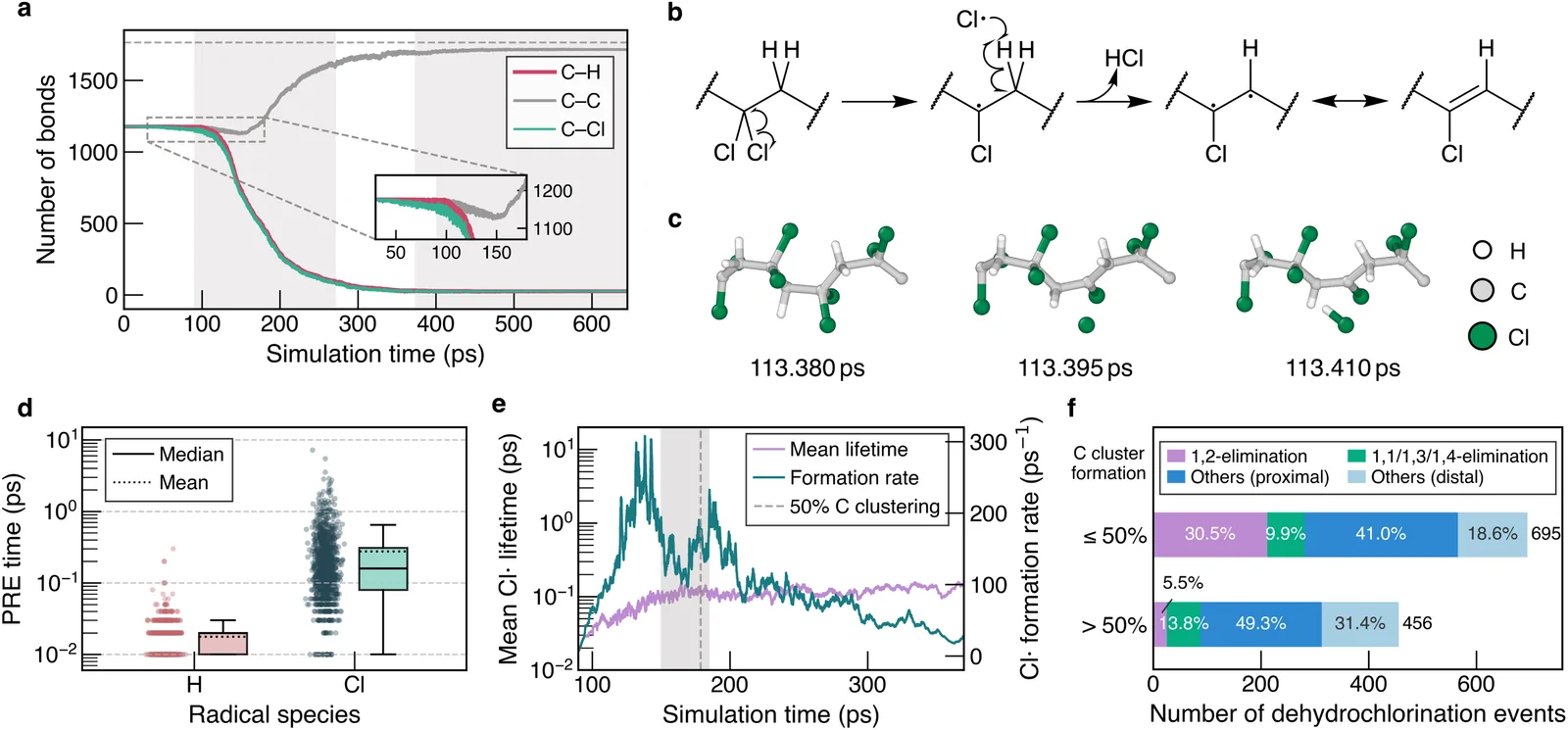
Radical-mediated dehydrochlorination mechanism. (a) Evolution of C–H (pink), C–C (grey), and C–Cl (green) bond counts during the simulation. The inset shows the 30–180 ps interval, highlighting that C–Cl cleavage precedes C–H cleavage, and that C–C scission occurs prior to carbon-network growth. (b) Schematic of the proposed radical dehydrochlorination mechanism, proceeding *via* homolytic C–Cl bond cleavage, hydrogen abstraction by Cl˙ radicals, and C–C π-bond formation. (c) Representative fragments from the simulation trajectory illustrating key steps of the proposed mechanism. (d) Pre-reactive-encounter (PRE) times of eliminated H (pink) and Cl (green) atoms. For each HCl-elimination event, the time is defined as the interval between the final detachment of the atom from the carbon framework and its first approach within 1.50 Å of any complementary species. Each dot represents an individual atom, with horizontal jitter added for visual clarity. Statistical information: box plots span the interquartile range (IQR), with solid and dotted lines denoting the median and mean, respectively; whiskers extend to 1.5× the IQR. (e) Evolution of the average lifetime (purple) and formation rate (green) of Cl˙ radicals defined on an event basis during the NVT* stages (90 ps to 370 ps). The point at which 50% of the C atoms have joined the largest connected carbon cluster is indicated by a dashed line, and the clustering period is shaded. (f) Regioselectivity of HCl elimination before and after 50% carbon-cluster formation. Events are classified according to the relative positions of the parent carbon atoms as 1,2-elimination (purple), 1,1/1,3/1,4-elimination (green), and other eliminations as described in the main text.

These observations are consistent with a radical-mediated dehydrochlorination mechanism, as illustrated schematically in Fig. 2b. The process is initiated by thermally induced C–Cl bond homolysis, which generates reactive Cl˙ radicals capable of abstracting hydrogen. When abstraction occurs from an adjacent carbon atom, HCl elimination is likely coupled to local C=C formation, as illustrated in Fig. 2b. However, if the Cl˙ radical migrates further before hydrogen abstraction, the resulting under-coordinated carbon sites are spatially separated from the original cleavage site and can participate in competing processes, including further chlorine detachment, radical migration, or termination through inter-chain C–C bond formation. Thus, Fig. 2b should be regarded as a simplified representation of a local HCl-forming pathway, rather than a complete description of all dehydrochlorination events observed during pyrolysis. Representative structural fragments extracted from the simulation trajectory, capturing key instances of these bond-level events, are shown in Fig. 2c. In the following trajectory analysis, the term “radical” is used operationally to denote a detached species identified based on bond connectivity, rather than an electronically characterized radical state: although the DFT computations used to generate MLIP training data were spin-polarized, the connectivity-defined radical species identified here do not necessarily imply persistent localized unpaired-spin states. However, radical involvement in PVDC dehydrochlorination has experimental precedent from ESR measurements, and is therefore not inferred solely from the present connectivity-based analysis.^29,30^

Fig. 2d shows the pre-reactive-encounter (PRE) times for isolated hydrogen and chlorine species. For each eliminated HCl molecule, we define this quantity as the time interval between the last instance at which either the hydrogen or chlorine atom is bonded to a carbon atom, and the subsequent moment this atom enters the reactive domain of a complementary species. Defining the time origin with respect to the last C–X (X = H or Cl) bond is necessary because radicals formed during the simulation frequently recombine to the carbon network before their final removal. In addition, the PRE time is intended to capture the duration for which a detached species remains isolated before its first reactive encounter; subsequent partner-exchange events prior to final HCl elimination are not included. Moreover, a more permissive H–Cl cutoff of 1.50 Å is used to define the reactive domain, rather than the 1.30 Å cutoff employed as the criterion for molecular removal. This separation of criteria accounts for high-temperature bond-length fluctuations, where H–Cl pairs may already be associated at thermally elongated separations before contracting below the removal cutoff.

The average PRE time for Cl˙ radicals is significantly longer than that for hydrogen, implying that the former persist as isolated species while diffusing to reactive hydrogen sites. In contrast, hydrogen atoms remained isolated for less than 0.02 ps on average, a timescale comparable to the period of a single C–H bond vibration (0.01 ps based on a wavenumber of 3000 cm^−1^).^53^ This likely suggests that isolated hydrogen atoms are not stabilized as independent radicals but exist only as transient states during abstraction. These disparate timescales support the proposed mechanism, in which the Cl˙ radicals act as the primary mobile species in the dehydrochlorination process.

To further characterize the temporal behaviour of Cl˙ radicals, we analysed their lifetime using an event-based algorithm. In this approach, each homolytic bond dissociation is treated as an independent event regardless of the atom's history: if a Cl˙ radical re-binds to the carbon network and subsequently detaches again, the newly formed radical is recorded as a separate data point. The resulting ensemble-averaged lifetimes are presented as a function of their generation time in purple in Fig. 2e. The average lifetime of Cl˙ radicals increases initially and stabilizes at approximately 0.10 ps around the 170 ps mark. This value is lower than the mean PRE time shown in Fig. 2d, as it also accounts for radical events that do not lead to HCl elimination.

The formation rate of Cl˙ radicals, as a function of their generation time, is shown by the green line in Fig. 2e. This quantity increases initially, reaching a maximum at 138.33 ps. Although this rate subsequently declines in tandem with the consumption of chlorine content, a secondary peak emerges at 185.22 ps, following a local minimum at 169.51 ps. This bimodal profile suggests that dehydrochlorination likely proceeds through two kinetically distinct stages under the imposed heating protocol. The later stage may involve elimination from already-unsaturated local environments, where higher activation barriers are accessed only at elevated temperature.

In general, the early stage of dehydrochlorination is characterized by rapid generation of Cl˙ radicals with relatively short lifetimes, whereas at later stages the radical population decreases while the average lifetime increases. This inverse relationship can be understood from the evolving regioselectivity of HCl elimination, as shown in Fig. 2f. The elimination events are first partitioned based on the time of the C–Cl cleavage into two categories: before and after the point at which 50% of the carbon atoms have joined the largest connected carbon cluster (indicated by the grey dashed line in Fig. 2e). The relative positions of the parent carbon atoms associated with each eliminated HCl pair are then computed and classified into 1,2-elimination, 1,1/1,3/1,4-elimination, or others. Although chlorine and hydrogen atoms are initially bonded to alternating carbon atoms in the precursor polymer, 1,1- and 1,3-eliminations occur due to frequent radical recombination and cross-linking, which alter the local atomic environment. These non-standard eliminations support the radical-mediated nature of the reaction. The “others” category, comprising long-range intra-chain and any inter-chain eliminations, is further subdivided based on the direct distance between parent carbon atoms. A threshold of 6.7 Å, corresponding to the mean carbon–carbon distance across all such events, is used to distinguish between proximal and distal eliminations.

Overall, the majority of dehydrochlorination events (60.4%) occurs prior to 50% carbon-cluster development. During this early stage, 1,2-elimination is one of the dominant pathways, favoured by the intrinsic proximity of adjacent reactive sites, which allows for efficient abstraction with minimal radical diffusion. Concurrently, the proportion of proximal inter-chain elimination is significant, which can be attributed to the crystalline packing of the precursor; specifically, the minimum inter-chain Cl⋯H distance of 3.22 Å is substantially shorter than the maximum 1,4-intra-chain distance of 6.20 Å, making inter-chain abstraction spatially favourable compared to long-range intra-chain events. This behaviour is consistent with the classical picture of competing zip-like intra-chain propagation and inter-chain radical transfer.^51^

As carbon cross-linking progresses, the proportion of 1,2-elimination diminishes in favour of distal and inter-chain pathways. Together with the higher Cl˙ lifetime (Fig. 2e) and apparent chlorine mobility at similar times (SI, Section S2.3), this shift suggests that HCl formation becomes increasingly encounter-limited as nearby H-containing sites are depleted and the carbon framework rigidifies.

### Carbon-network formation

Having examined the mechanisms of dehydrochlorination, we now turn to the structural evolution of the carbon network. Fig. 3a depicts the temporal evolution of carbon atoms categorized by their coordination numbers (CNs). Notably, an initial decrease in the population of fourfold-coordinated carbon is observed before the first HCl-removal event at 100 ps. We attribute this structural change to the homolytic cleavage of C–Cl bonds, the accumulation of which sets the stage for subsequent dehydrochlorination. Beyond 100 ps, the rapid conversion of fourfold- to threefold-coordinated carbon atoms reflects the accelerating rate of dehydrochlorination.

**Fig. 3:**
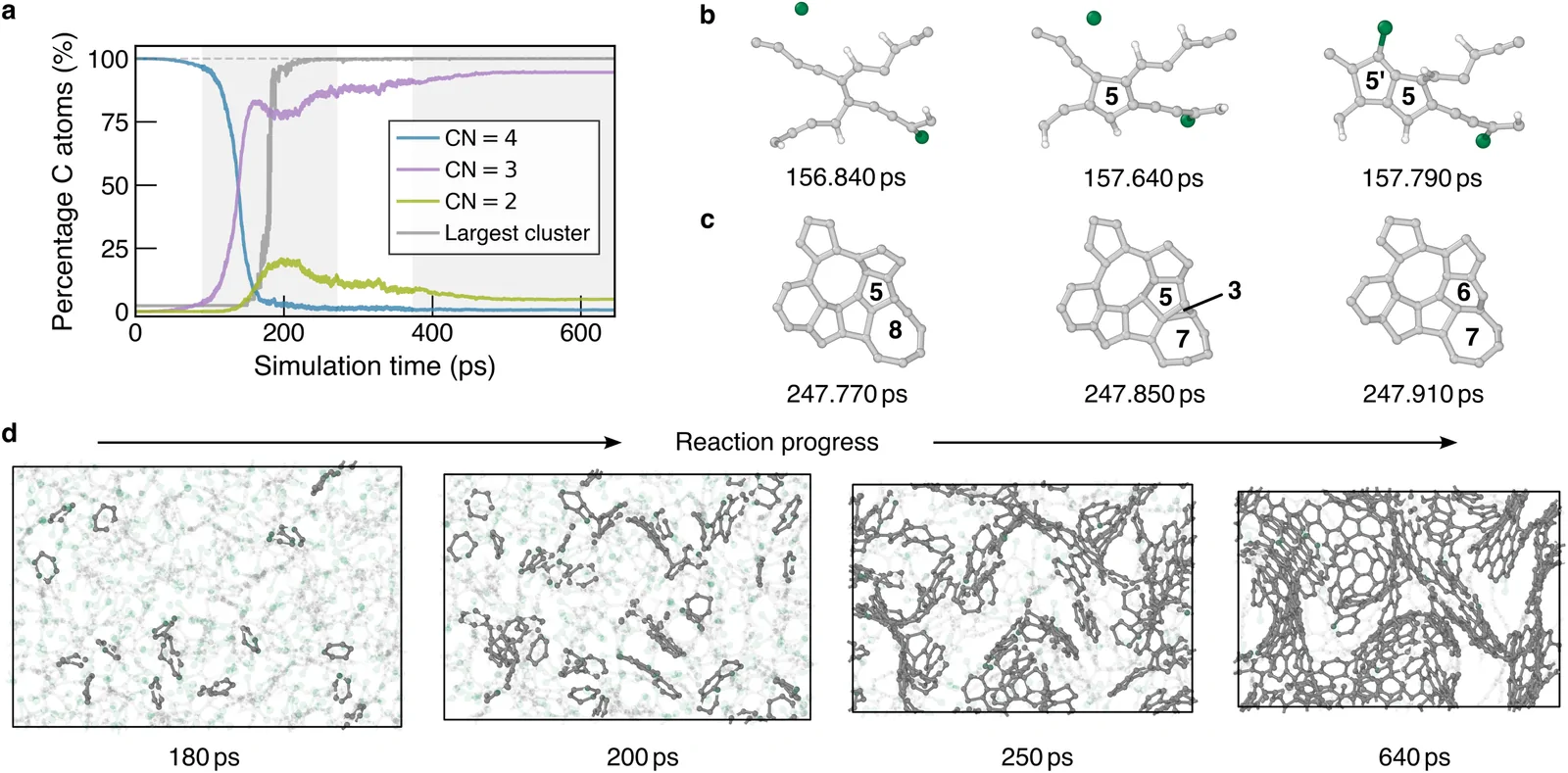
Carbon network formation and rearrangement. (a) Evolution of the relative abundance of carbon atoms categorized by coordination number (CN): four (blue), three (purple), and two (green). The grey line tracks the fraction of carbon atoms belonging to the largest cluster, defined as the largest set of carbon atoms linked through continuous C–C bonds. (b) Representative fragments illustrating the stepwise cross-linking of unsaturated carbon chains and the resulting ring formation. (c) Representative fragments illustrating the rearrangement of five- and eight-membered rings into six- and seven-membered rings, *via* a three-membered ring intermediate. (d) Structural snapshots highlighting six-membered rings to track their spatial development throughout the simulation. Frames are drawn to scale with the simulation cell.

Given that each carbon atom in pristine PVDC is bonded to two substituents, continued dehydrochlorination would be expected to drive a further transition from threefold- to twofold-coordinated states. Indeed, the CN = 3 population initially rises to 83.6% at 158.77 ps, then declines to 75.9% at 196.32 ps, where the CN = 2 population reaches its maximum. This transition from threefold- to twofold-coordinated carbon coincides with the second maximum in the Cl˙ formation rate, observed at 185.22 ps (Fig. 2e). However, most of these CN = 2 sites are quickly consumed, as their appearance is followed by a sharp rise in the size of the largest carbon cluster, defined as the maximal set of carbon atoms connected *via* continuous C–C bonds. This suggests simultaneous C–C network development, where these high-energy twofold-coordinated intermediates are rapidly incorporated into the growing network, restoring a more stable sp^2^-rich state. The MACE-predicted per-atom energy contributions for carbon atoms with different local CN are given in Section S2.6 of the SI, where twofold-coordinated carbon atoms are shown to be associated with the highest local energies among the coordination environments analysed.

Selected structural fragments in Fig. 3b illustrate the cross-linking of carbon chains through a stepwise mechanism mediated by twofold-coordinated carbon intermediates. These under-coordinated sites are highly reactive and trigger successive C–C coupling events between neighbouring unsaturated sites. The time separation between successive C–C bond-formation events is on the order of picoseconds, much longer than typical C–C vibrational periods (20 fs to 50 fs for stretching modes and up to 100 fs for bending modes),^53^ indicating that ring closure proceeds stepwise rather than in a concerted fashion. We also note that the Cl˙ radical visible in the upper left of the first two frames in Fig. 3b recombines with the carbon network in the final frame, exemplifying a radical-addition event that occurs frequently throughout the simulation.

Although carbon-network aggregation is rapid and reaches a plateau by 185.17 ps, the structural snapshot at around this stage (first frame in Fig. 3d) contains only a sparse population of six-membered rings. This observation suggests that, although the carbon scaffold assembles rapidly, aromatic ordering continues through subsequent internal rearrangement. The evolution of five- to seven-membered ring populations (Fig. S7a) shows that five- and six-membered rings form rapidly at early stages of the simulation, whereas seven-membered rings emerge more slowly. We also note a slight decrease in the seven-membered-ring population and a continued increase in six-membered rings after five-membered-ring formation has saturated, suggesting continued annealing of larger non-hexagonal motifs into more stable six-membered rings during early quenching. One such pathway is shown in Fig. 3c, where a fused five- and eight-membered ring pair undergoes stepwise conversion into fused six- and seven-membered motifs, *via* a three-membered-ring intermediate.

However, Fig. S7a also suggests that five- and seven-membered rings remain substantial in the final structure even after extensive quenching. Fig. 3d, which highlights six-membered rings in the trajectory snapshots, shows their progressive growth towards the end of pyrolysis within a curved network. The correlation between local ring topology and curvature is quantified in Fig. S7c, where threefold-coordinated carbon atoms participating in fewer six-membered rings exhibit larger out-of-plane distances. In addition, these threefold-coordinated carbon atoms are also associated with higher atomic energies (Fig. S9b) – although the energetic penalty appears to be modest enough to allow them to persist in the final structure. Taken together, these results suggest that five- and seven-membered rings are not merely transient defects, but are incorporated into the evolving carbon framework in a way that preserves local curvature and frustrates complete graphitic ordering, consistent with previous experimental observations of non-hexagonal defects in non-graphitizing carbon.^35,36^

## Conclusions

We have developed a bespoke MLIP model and a density-calibrated simulation protocol to investigate the atomistic mechanisms of hard carbon formation from PVDC decomposition. Our results show that the carbonization stage of pyrolysis proceeds *via* thermally induced, radical-mediated dehydrochlorination, initiated by C–Cl bond cleavage and the formation of reactive Cl˙ species. This mechanism is similar to that proposed by Davies *et al.* on the basis of kinetic measurements,^51^ and is compatible with recent quantum-mechanical nanoreactor simulations of PVC pyrolysis that likewise identified chlorine-radical-initiated hydrogen abstraction.^54^ The regioselectivity of subsequent hydrogen abstraction evolves with the progress of pyrolysis, with Cl˙ radicals diffusing over longer distances to react as the network matures. In particular, we identify secondary elimination from already-unsaturated sites as a key process in driving a stepwise carbon cross-linking process. This leads to the formation of a defect-rich carbon network, which is subsequently annealed through internal rearrangement. The persistence of five- and seven-membered rings supports the long-standing view that non-hexagonal defects introduce curvature into graphene-like fragments.^13,26,33,34,36^ Similar defects have been incorporated into, or observed in, atomistic models of non-graphitizing carbon.^41,42,55–60^ Our work complements these experimental and simulation studies by providing an MLIP-driven atomistic pathway for the defect formation during PVDC pyrolysis.

These mechanistic insights suggest a possible origin of the contrasting carbonization behaviour of PVDC and PVC. The stoichiometric H/Cl balance of PVDC favours extensive HCl elimination and the formation of a carbon-rich residue, which may promote early C–C cross-linking and kinetically trap the disordered, non-hexagonal motifs observed here.^18,26–28,51^ In contrast, PVC retains excess hydrogen after dehydrochlorination, and the resulting hydrogen-rich phase undergoes continued rearrangement with the evolution of hydrocarbons and tar.^18,52,54^ Notably, PVC carbonization has been reported to pass through a viscous phase, providing structural mobility for developing aromatic domains to rearrange before the network becomes rigid.^18,31,50^ However, as the present simulations consider only PVDC, whether these differences underlie the contrasting graphitizability of the two precursors remains to be established.

Two directions for further study therefore emerge. First, MLIP-driven simulations offer a compelling opportunity to systematically compare PVC and PVDC pyrolysis, which could provide an atomistic basis to revisit Franklin's foundational classification of hard and soft carbon materials.^16–18^ Although the MLIP model developed in our work contains the necessary chemical elements, a quantitative PVC study would require additional training and validation on PVC-specific environments. Furthermore, the MD protocol would need to be adapted to accommodate the more complex volatile-product chemistry of PVC degradation, since hydrogen and chlorine are no longer present in stoichiometric balance, and HCl is therefore no longer expected to be the only dominant volatile product. Second, the carbon structures generated here could serve as realistic starting points for simulating alkali-metal insertions, extending beyond prior MLIP-driven studies that relied on melt-quench-derived proxies,^58–60^ and complementing the experimentally-informed approach of ref. 61. Recent computational studies have begun to investigate hard carbons derived from other precursors: first-principles simulations were reported for lignin-derived hard carbons as anode materials for Li-, Na-, and K-ion batteries,^62^ and experimentally-informed MD simulations were carried out to study hard carbons formed from nanocellulose.^61,63^ We anticipate that the MLIP-driven workflow developed here can be extended to these chemically more complex precursor systems, enabling simulations at larger length and time scales and facilitating direct comparisons between precursor chemistry, hard-carbon structure, and electrochemical performance. In a wider context, our study illustrates the growing role of atomistic ML methods in understanding the chemical mechanisms of the synthesis of structurally complex functional materials, paving the way for predictive simulations that guide the design of amorphous materials at the atomic level.^64^

## Methods

### Dataset

The final MLIP model was trained on a dataset comprising 3725 structures (426 570 atoms). Most PVDC-pyrolysis configurations were generated through active learning. A total of 1172 structures were produced using the CASTEP + ML framework, following ref. 65. This approach accelerates *ab initio* MD simulations by fitting Gaussian Approximation Potential (GAP)^66^ models on the fly, with uncertainty-based criteria determining whether forces are evaluated using DFT or ML predictions; this framework has been used previously in the construction of a dataset and MLIP model for graphene oxide.^67^ An additional 956 PVDC-based structures were sampled from iterative MD trajectories. These simulations were performed on 216- or 192-atom supercells derived from single-crystal XRD data^49^ and encompassed a broad range of thermodynamic conditions: (i) NVT simulations up to 3000 K; (ii) NPT simulations at 1 bar and up to 1800 K; (iii) NPT compression simulations spanning 1000 K to 3000 K and 10 GPa to 150 GPa; and (iv) trial pyrolysis simulations. To improve coverage of molecular by-products, the dataset was augmented with 170 chlorinated hydrocarbon molecules from PubChem^68^ and their distorted variants, as well as 358 SOAP^69^-selected QM9 (ref. 70) molecules featuring random Cl-for-H substitutions with scaled bond lengths. The description of bulk carbon phases was reinforced with 450 amorphous carbon,^71^ 162 graphite,^71^ and 457 C–H^72^ configurations from previous studies.

### DFT computations

All structures in the dataset were relabelled for consistency. Single-point energies, forces, and stresses were computed using density functional theory (DFT) as implemented in CASTEP 24.1,^73^ with the Perdew–Burke–Ernzerhof (PBE) generalized-gradient approximation^74^ and Tkatchenko–Scheffler (TS) semi-empirical dispersion corrections.^75^ The plane-wave cutoff energy was set to 700 eV, with a self-consistent field convergence threshold of 10^−7^ eV. To accurately describe open-shell species generated during bond dissociation, spin-polarized computations were performed; an initial magnetic moment of 1*µ*_B_ was assigned to each atom and allowed to relax freely to facilitate convergence. The Brillouin zone was sampled using Monkhorst–Pack grids^76^ with a maximum *k*-point spacing of 2π × 0.04 Å^−1^. Core electrons were described using on-the-fly-generated pseudopotentials in CASTEP.

### MLIP model

We trained a bespoke MACE potential model^48^ to describe the thermal decomposition of PVDC. The dataset was partitioned by assigning 10% of each configuration type to the test set and a further 10% of the remaining structures to the validation set. We employed a ScaleShiftMACE architecture comprising two message-passing layers with 128 equivariant channels and equivariant features up to *l* = 1. Each interaction block used a correlation order of three and included angular channels up to *l*_max_ = 3, with a cutoff radius of *r*_max_ = 6.0 Å. Training was conducted using a standard weighted energy-forces loss function, with loss weights set to *λ*_E_ = 1, *λ*_F_ = 100 for the first 150 epochs, and adjusted to *λ*_E_ = 1000, *λ*_F_ = 100 for the final 100 epochs. The model was trained using the Adam optimizer^77^ with a batch size of 6, an initial learning rate of 0.01, a stochastic weight-averaging (SWA) learning rate of 0.001, and weight decay of 5 × 10^−7^. While the MACE model was trained on spin-polarized DFT calculations with magnetic moments converged self-consistently, we acknowledge that alternative spin configurations were not systematically explored for each geometry, and the MLIP does not explicitly resolve spin states or transitions between them. Therefore, spin-related effects, such as singlet–triplet crossings during radical reactions, are not explicitly captured. On the test set, our final model achieved root-mean-square errors of 6.9 meV at^−1^ (0.67 kJ mol^−1^) for energies and 269.6 meV Å^−1^ for forces. All training was performed on a single RTX 6000 Ada GPU.

### MD simulations

MD simulations were performed using LAMMPS^78^ with the MACE/kk pair style in KOKKOS mode on a single GPU. The simulation protocol is summarized in Fig. 1b and discussed in the Results section. To maintain numerical stability and resolve high-temperature atomic motion, the integration timestep was adjusted according to the simulation stage: 0.5 fs for the NPT heating, 0.25 fs for the two NVT* stages, and 1.0 fs for the NPT* quenching. A Nosé–Hoover thermostat and barostat^79^ were employed, with temperature and pressure damping constants set to 0.1 ps and 1.0 ps, respectively. The initial simulation supercell comprised 42 parallel PVDC chains (3528 atoms), with dimensions of 47.0 Å × 34.0 Å × 31.5 Å. Periodic boundary conditions were applied in all three spatial directions.

Starting from the second stage, segmented MD simulations were performed using a custom Python workflow, which automated periodic HCl removal, cell rescaling, and the initiation of successive simulation segments. The NVT* stages and the final NPT* quench were partitioned into segments of 1.0 ps and 10.0 ps, respectively. During each segment, the simulation advanced according to the programmed temperature ramp, isothermal hold, or quench. Between successive segments, the simulation was paused to identify and remove HCl molecules within a bonding cutoff of 1.30 Å. To maintain a physically realistic density following the volatile loss, the simulation cell and the atomic coordinates were isotropically rescaled. The updated structure was written to a LAMMPS data file along with velocities and used to initialize the subsequent segment.

The volume contraction associated with the removal of each HCl molecule was estimated to be around 29.7 Å^3^ from the sum of the spherical van der Waals volumes of H (*r*_vdW_ = 1.20 Å) and Cl (*r*_vdW_ = 1.75 Å) atoms.^80^ We recognize, however, that the volume reduction associated with HCl removal in a condensed pyrolyzing solid is not necessarily captured by the sum of isolated van der Waals volumes; this quantity was thus treated as an empirical parameter and was calibrated against experimental density data. A value of 30.0 Å^3^ per removed HCl molecule was adopted, as it yielded good agreement with reported densities for PVDC-derived hard carbons. For comparison, a larger value of 33.5 Å^3^ was also tested, which resulted in a higher final density and an increased fraction of threefold-coordinated carbon atoms, while preserving the qualitative features of the pyrolysis process.

A detailed analysis of the sensitivity of the simulated pyrolysis pathway to the density-rescaling parameter and the maximum annealing temperature can be found in Section S1.3 of the SI. While these parameters affect the final density and extent of HCl elimination, all tested protocols produce carbon networks with similar coordination environments and ring populations.

### Structural analysis

Bond statistics, atomic coordination, and carbon cluster size were determined from neighbour-list analysis as implemented in the Atomic Simulation Environment (ASE) package,^81^ using element-specific cutoffs of 1.30 Å (C–H), 1.85 Å (C–C), and 2.10 Å (C–Cl). The development of the carbon cluster was quantified by constructing a connectivity graph for each trajectory frame, in which carbon atoms were treated as nodes and C–C bonds as edges. The size of the largest connected component was then extracted from this graph. Six-membered rings were identified using a minimum cycle basis algorithm on the carbon connectivity graph; carbon atoms belonging to these hexagonal rings were flagged. For visualization, non-ring atoms were rendered with increased transparency using the Compute Property modifiers in OVITO.^82^

### Radical analysis

Atomic indices changed due to variations in atom count during the simulation. To enable time-resolved analysis, atoms were assigned persistent identifiers based on their indices in the initial frame. After each HCl-removal step, remaining atoms were remapped across subsequent frames using position matching under periodic boundary conditions. Pre-reactive-encounter times were determined by back-tracing eliminated atoms from the point of removal to the last frame in which they were covalently bonded to a carbon atom. A “reactive encounter” was defined as the first subsequent frame where the radical came within 1.50 Å of a complementary species.

In the event-based radical lifetime analysis, chlorine atoms lacking neighbours within expanded cutoffs of 1.60 Å (H–Cl), 2.30 Å (C–Cl), 2.40 Å (Cl–Cl), were classified as radicals. For each chlorine atom, consecutive frames satisfying this criterion were grouped into a single radical event, from which the formation time and lifetime were extracted. A rolling mean was applied to the lifetime data to reduce noise.

For each elimination event, the parent carbon sites – those previously bonded to the eliminated hydrogen and chlorine atoms – were identified using the established bonding cutoffs. The shortest-path separation between these parent sites at the point of detachment was quantified using a topological C–C connectivity metric, and elimination events were classified accordingly. For long-range or inter-chain cases, the spatial proximity was further characterized by Euclidean distances under periodic boundary conditions.

## Author contributions

L. W. and V. L. D. designed the study. L. W. carried out the main computational work, data analysis, and visualization. Z. W. performed pilot studies with guidance from Z. E.-M. and contributed to developing a subset of the training data. L. W. and V. L. D. wrote the manuscript with input from Z. E.-M.; V. L. D. supervised the work. All authors approved the manuscript submission.

## Conflicts of interest

There are no conflicts to declare.

## Supplementary Material

SC-OLF-D6SC05974K-s001

## Data Availability

Data supporting this study, including the MLIP parameters and relevant structural data, are openly available at https://github.com/vldgroup/papers-pvdc. Supplementary information (SI): MLIP validation and simulation-protocol sensitivity tests, structural characterization, molecular-product statistics, and additional analyses of reaction mechanisms and carbon-network topology. See DOI: https://doi.org/10.1039/d6sc05974k.
