# Supplementary material for: Atomistic mechanisms of hard carbon formation from polyvinylidene chloride
Source: Chem Sci. 2026 Oct 8. Online ahead of print. doi: 10.1039/d6sc05974k (PMC13714722; doi:10.1039/d6sc05974k)
Supplement: Supplementary file 1 [file SC-OLF-D6SC05974K-s001.pdf]

Supplementary Information (SI) for Chemical Science.
This journal is © The Royal Society of Chemistry

Electronic Supplementary Information (ESI) for

"Atomistic Mechanisms of Hard Carbon Formation from Polyvinylidene Chloride"

Litong Wu[1], Zitong Wu[1], Zakariya El-Machachi[1], and Volker L. Deringer*[1]

[1]*Inorganic Chemistry Laboratory, Department of Chemistry, University of Oxford, Oxford, UK*

Contents

S1 Methods **S2**
- S1.1 Dataset . . . S2
- S1.2 MLIP model validation . . . S3
- S1.3 MD sensitivity test . . . S5
- S1.4 Final structure characterization . . . S7

S2 Results **S9**
- S2.1 Molecular product statistics . . . S9
- S2.2 Kinetic analysis of dehydrochlorination . . . S10
- S2.3 Diffusivity analysis . . . S11
- S2.4 Evolution of local coordination environments . . . S12
- S2.5 Topological evolution . . . S14
- S2.6 Atomic-energy descriptors of different local environments . . . S16
- S2.7 Structural signatures of under-coordinated carbon intermediates . . . S19

S3 Supplementary references **S23**

*volker.deringer@chem.ox.ac.uk

S1 Methods

S1.1 Dataset

The composition of the complete dataset is summarized in Table S1. For each configuration type, 10% of the structures were reserved as the test set and used for the numerical tests in Figure S1a,b. The remaining structures were partitioned into training and validation sets in a 9:1 ratio.

The dataset was designed to span the chemical environments encountered during PVDC pyrolysis, from intact and decomposing polymer configurations to chlorinated molecular fragments, hydrogenated carbon networks, amorphous carbon, and graphitic configurations. Here, PVDC-1 denotes configurations generated using the CASTEP+ML approach,[S1] whereas PVDC-2 denotes configurations generated from iterative MLIP-driven MD. Some configurations are taken from previous work,[S2–S5] as indicated in Table S1. Detailed descriptions of each configuration type are provided in the Dataset subsection of the Methods section in the main text.

Table S1: Composition of the PVDC dataset (including the test set), with the number of structures, total atom counts, element-resolved atom counts, and sources for each configuration type.

**Config types**	**Structures**	**Atoms**	**Elements**			**Sources**
			**C**	**H**	**Cl**	
Isolated atoms	3	3	1	1	1	
PVDC-1	1,303	116,904	38,968	38,968	38,968	CASTEP+ML[S1]
PVDC-2	1,064	174,248	75,944	49,238	49,066	Iterative MD
Amorphous C	500	100,000	100,000	0	0	Rowe *et al.*[S2]
Graphite	180	6,324	6,324	0	0	Rowe *et al.*[S2]
CH configurations	509	66,938	28,930	38,008	0	Ibragimova *et al.*[S3]
C/H/Cl molecules	189	2,322	1,130	853	339	PubChem[S4]
QM9 molecules	398	7,584	3,262	4,029	293	QM9[S5]
**Total**	**4,146**	**474,323**	**254,559**	**131,097**	**88,667**	

S1.2 MLIP model validation

The MACE[S6] model fitted to the final dataset was validated using both global test-set errors and targeted checks relevant to the chemical and structural environments encountered during PVDC pyrolysis. A parity analysis assesses overall interpolation accuracy across the labelled dataset, while additional tests examine transferability to bond elongation, carbonized structures, and high-temperature dynamics.

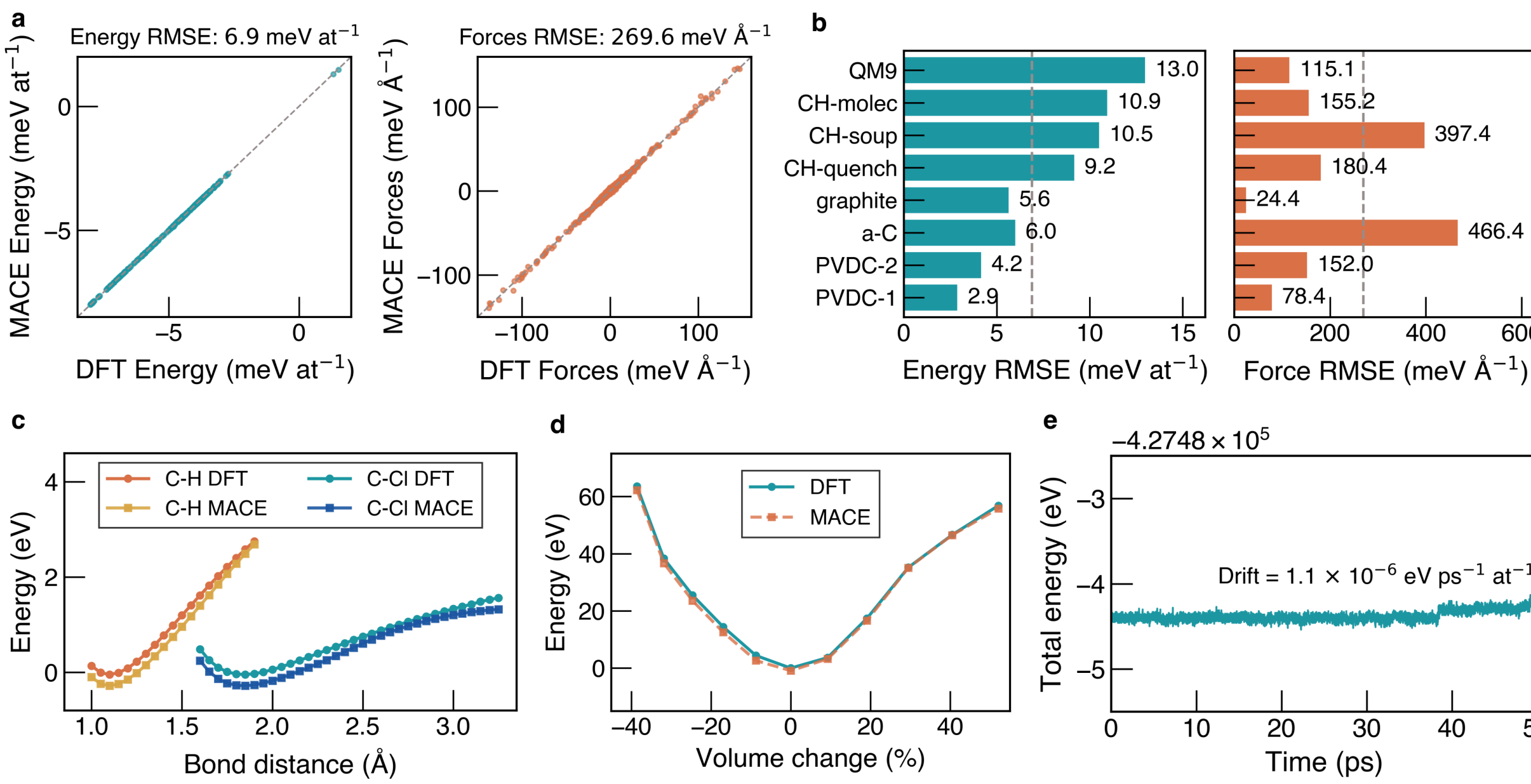

Figure S1: Numerical validation and robustness tests of the MACE model. (**a**) Parity plots comparing MACE-predicted and DFT-computed per-atom energies (*left*) and forces (*right*) for the test set. Root-mean-square errors (RMSEs) are indicated above each panel. (**b**) Per-atom energy (*left*) and force (*right*) RMSEs resolved by configuration type. Gray dashed lines indicate the corresponding average RMSEs over the full test set. (**c**) MACE- and DFT-computed bond-stretching energy profiles for randomly selected C–H (*orange*) and C–Cl (*blue*) bonds in a single 48-atom PVDC chain. The C–H bond was stretched over 1.0 Å to 1.9 Å; C–Cl bond, 1.6 Å to 3.3 Å, in increments of 0.05 Å. At each sampled bond length, spin-polarized DFT geometry optimizations were performed with the selected bond length and simulation-cell dimensions held fixed. Energies are reported relative to the DFT energy of the original structure. (**d**) Equation-of-state (EoS) validation of a 250-atom pyrolysed structure obtained from a small-scale MD simulation following the production protocol. The simulation cell was isotropically scaled over 11 volumes spanning compressed and expanded states relative to the reference structure, after which atomic positions were optimized with the BFGS optimizer[S7–S10] in ASE using the MACE model, until the maximum force was below 0.01 eV Å^{-1}. MACE energies are compared with DFT energies for the corresponding MACE-optimized structures; energies are reported relative to the original structure. (**e**) Microcanonical (NVE) stability test for a 2160-atom PVDC structure initialized at 3000 K and propagated for 50 ps.

The global parity plots in Figure S1a show that the model reproduces the DFT-labelled test set with low energy error and acceptable force error across the diverse chemical environments included

in the dataset. The configuration-resolved errors in Figure S1b show systematic dependence on the type of local environment sampled. Energy errors are relatively larger for the molecular subsets, where the smaller number of atoms per structure reduces the averaging effect present in bulk configurations. In contrast, the largest force errors occur for the CH "soup" (see Ref. S3) and amorphous-carbon (see Ref. S2 and references therein) configurations, which contain highly disordered and high-energy local environments. These environments produce a wider force distribution, making accurate force prediction intrinsically harder.

The constrained bond-stretching scans in Figure S1c test the local description of C–H and C–Cl bond elongation, which is directly relevant to the simulated dehydrochlorination mechanism. The close agreement between MACE and DFT energies along these scans indicates that the model captures the energetic response to bond distortions well. Since only one C–H bond and one C–Cl bond were selected along the polymer chain, this test should be viewed as a targeted validation of bond-elongation behaviour rather than a reaction-path benchmark.

The EoS comparison in Figure S1d assesses the model's ability to describe a dense carbonized structure representative of the later stages of pyrolysis. Agreement between MACE and DFT energies over compressed and expanded configurations indicates that the model remains reliable beyond PVDC-like environments and can describe the structural response of a carbon-rich network.

The NVE test in Figure S1e assesses the numerical stability and energy conservation of the model under high-temperature conditions. In an ideal NVE simulation, the total energy should remain constant throughout the trajectory; in practice, numerical integration errors and inaccuracies in the underlying potential-energy surface (PES) can accumulate over time, leading to artificial energy drift. The very small drift of approximately $1.1 \times 10^{-6}\,\mathrm{eV\,atom^{-1}\,ps^{-1}}$ observed over 50 ps at 3000 K therefore indicates stable numerical integration and a smooth MACE PES under high-temperature conditions.

S1.3 MD sensitivity test

To assess how sensitive the simulated pyrolysis pathway is to the density-rescaling parameter and the maximum annealing temperature, two additional simulations were performed and compared with the reference production trajectory. Test 1 used the same temperature schedule as the reference simulation but a larger volume decrement of 33.5 Å^3 per removed HCl molecule, compared with 30.0 Å^3 in the reference case. Test 2 used the same volume decrement as the reference simulation but a lower maximum temperature of 2500 K.

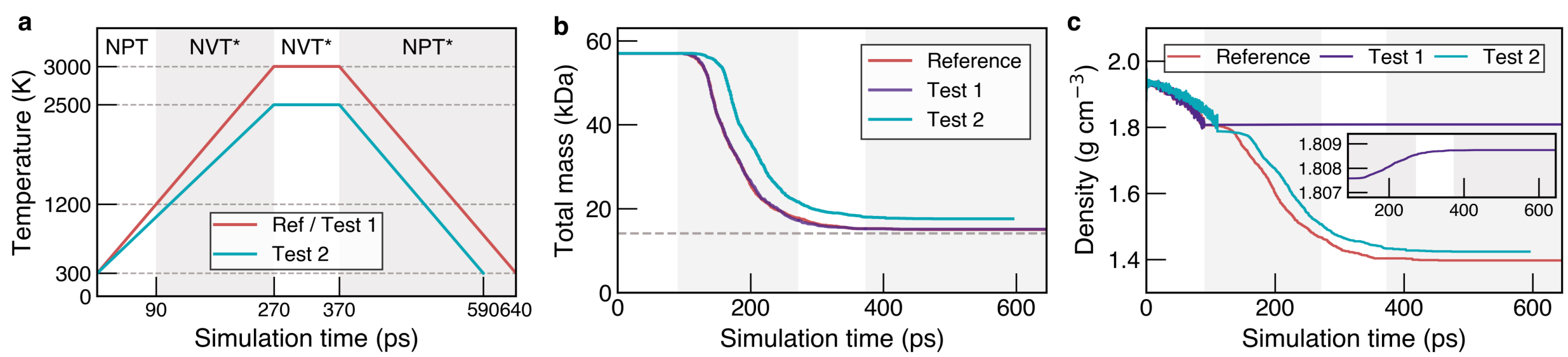

Figure S2: Sensitivity of PVDC pyrolysis to simulation parameters. (**a**) Temperature profiles used for the reference simulation/Test 1 (*pink*) and Test 2 (*blue*). The reference simulation and Test 1 followed the same temperature schedule; Test 2 used a lower maximum temperature of 2500 K and a reduced heating rate to maintain the same heating duration, while the quenching rate was kept constant. (**b**) Cumulative mass change associated with HCl removal. The gray dashed line marks the mass expected after complete HCl elimination. (**c**) Density evolution for the three simulations. The inset highlights the small density variation observed in Test 1 after the NPT stage.

As shown in Figure S2b, the reference simulation and Test 1 show nearly identical mass-loss profiles, indicating that the larger volume decrement has little effect on the simulated dehydrochlorination rate. As expected, however, Test 1 reaches a substantially higher final density as shown in Figure S2c because each HCl-removal event is followed by a larger cell-volume reduction. This comparison therefore separates the chemical progress of dehydrochlorination from the density imposed by the rescaling procedure.

By contrast, lowering the maximum temperature in Test 2 delays the onset of dehydrochlorination and leads to a lower overall mass loss (Figure S2b), implying less complete HCl elimination. As seen in Figure S2c, the density decrease is also delayed, and the final density remains slightly higher than that of the reference simulation. This is likely because the lower extent of HCl removal leaves more mass in the simulation cell, partly offsetting the density decrease associated with cell rescaling. Thus, the volume decrement primarily controls the final density, whereas the maximum

temperature primarily affects the extent and timing of HCl elimination.

Final-state structural metrics are summarized in Table S2. Carbon coordination was computed using the same neighbour-list procedure as described in the Methods section of the main text. Ring statistics were computed from a carbon-only connectivity graph constructed using a C–C cutoff of 1.85 Å. Rings were identified using a shortest-path ring-search algorithm[S11] on the carbon connectivity graph: for each C–C edge, the edge was temporarily removed and the shortest alternative path between the two bonded carbon atoms was identified; the removed edge together with this path defines the ring associated with that edge. Ring abundances are defined as the percentage of unique carbon atoms participating in at least one *n*-membered ring. Because an atom may belong to multiple rings in fused networks, the percentages are not mutually exclusive, and should be interpreted as internally consistent comparative metrics rather than unique topological decompositions.

Table S2: Final-state properties from the MD sensitivity tests.

		**Reference**	**Test 1**	**Test 2**
T_{max} (K)		3000	3000	2500
ΔV_{HCl} (Å^3)		30.0	33.5	30.0
HCl elimination (%)		97.9	97.7	90.6
Final density (g cm^{-3})		1.40	1.81	1.42
C coordination number (%)	2-fold	4.8	1.1	5.7
	3-fold	94.6	97.4	92.9
	4-fold	0.6	1.4	1.4
C ring abundance (%)	5-membered	50.9	57.2	51.3
	6-membered	80.4	81.2	70.7
	7-membered	25.3	38.0	24.5

All three simulations produce predominantly threefold-coordinated carbon networks, despite differences in final density and percentage HCl elimination. Increasing the volume decrement slightly increases the fraction of threefold-coordinated carbon, consistent with the higher final density favouring a more connected carbon network.

The ring statistics further indicate that non-hexagonal motifs are retained across all tested protocols. Although the precise distribution of ring sizes varies, five- and seven-membered rings remain present in all final structures. This supports the conclusion that odd-membered-ring formation is not an artefact of the reference density-rescaling parameter or maximum temperature.

S1.4 Final structure characterization

To validate the physical outcome of the model and MD scheme, the final hard-carbon product was characterized in real and reciprocal space, and compared with graphite and experimental PVDC-derived carbons. The C–C radial distribution function (RDF), $g_{CC}(r)$, was calculated, with reference C–C pair distances in graphite indicated below.[S12] An X-ray diffraction pattern was also simulated from the partial RDF using Faber–Ziman partial structure factors and X-ray atomic form factors for Cu Kα_1 radiation.

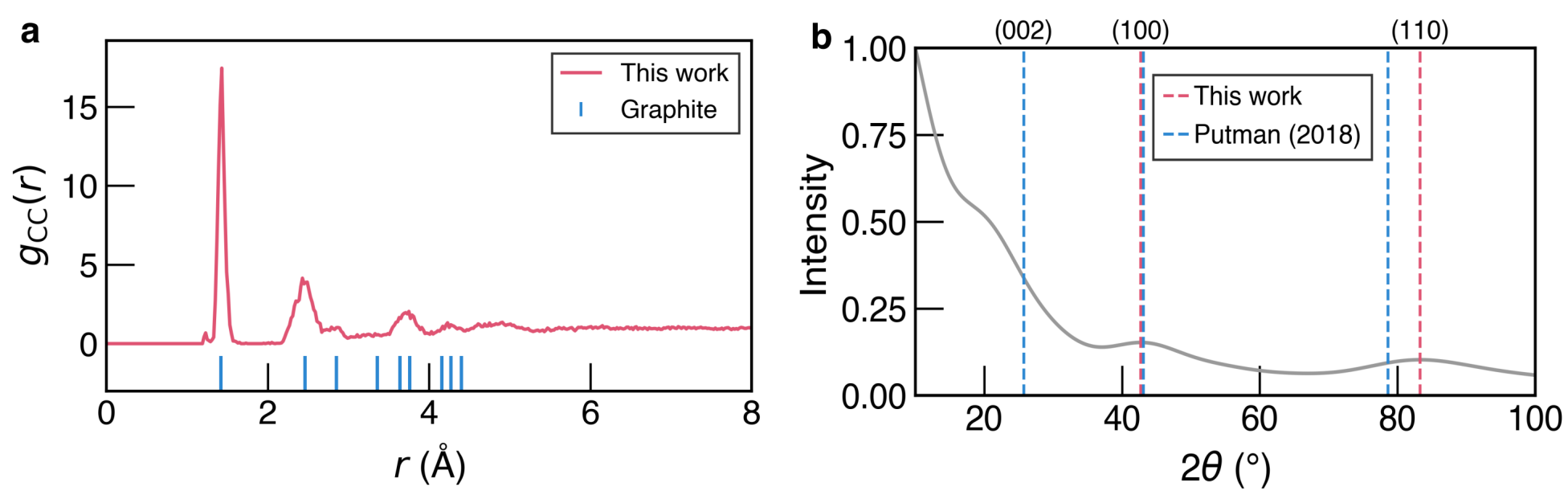

Figure S3: Structural characterisation of the PVDC-derived carbon. **(a)** C–C radial distribution function of the simulated hard carbon. Blue markers indicate C–C pair distances in graphite up to 4.5 Å.[S12] **(b)** Simulated X-ray scattering intensity for Cu Kα_1 radiation. Pink and blue dashed lines indicate the simulated and experimental band positions, respectively, for PVDC-derived carbon treated at 3000 °C from Putman *et al.*[S13]

The RDF in Figure S3a shows graphene-like short-range order: the first C–C peak and subsequent maxima broadly coincide with graphite pair distances.[S12] At larger distances, the correlations broaden and $g_{CC}(r)$ approaches unity, indicating limited long-range crystalline order. This is consistent with the simulated diffraction pattern in Figure S3b. The (100) maximum occurs at $2\theta = 42.7°$, very close to the 43.1° maximum of PVDC-derived carbon treated at 3000 °C.[S13] However, the simulated (110) maximum is shifted to a slightly higher angle, and only a weak shoulder is present in the (002) region rather than a well-resolved reflection.

Recognizing the finite-size limitations of the final 1226-atom cell, additional real-space analyses were performed (Table S3) to distinguish local structural order from longer-range correlations. Graphene-like domains were identified as connected patches of six-membered carbon rings using a 1.85 Å C–C cutoff. The lateral size of each domain was estimated by calculating the total area occupied by its six-membered rings, using the graphite ring area of 5.26 Å^2, and converting this area

Table S3: Real-space structural characteristics of the simulated and experimental PVDC-derived carbons.

	This work	1000 °C	3000 °C
L_a (Å)	15.2 ± 0.7	16[S14]	50[S14]
Interlayer separation (Å)	3.62 ± 0.28	3.67[S13]	3.46[S13]

to the diameter of an equivalent circular domain. The ring-weighted mean L_a was 15.2 Å, comparable to Franklin's 16 Å at 1000 °C, but substantially smaller than the 50 Å reported at 3000 °C.[S14] However, even the longest dimension of the simulation cell is only 33.27 Å, limiting the formation of independent graphene-like domains approaching the experimentally observed length scale.

Interlayer separation was measured directly between locally parallel graphene-like regions. Rings were considered stacked when their normals differed by less than 20°, their lateral offset was below 2.5 Å, and their separation along the mean ring normal lay between 3.3 Å and 4.5 Å. The resulting mean separation of 3.62 Å lies between the experimental values of 3.67 Å and 3.46 Å at 1000 °C and 3000 °C, respectively.

Overall, the simulated carbon reproduces the local structural characteristics of experimental PVDC-derived carbon, while medium- and long-range ordering remains limited due to the limited dimensions of the simulation cell. As the present work focuses on the atomistic mechanisms of hard-carbon formation during PVDC pyrolysis, the good local agreement supports the relevance of the simulated structures.

S2 Results

S2.1 Molecular product statistics

To quantify the relative abundance of molecular products formed during the production trajectory, we identified H_2, HCl, and Cl_2 molecules using distance-based bonding criteria and a minimum lifetime threshold of 0.01 ps. The bonding cutoffs, listed in Table S4, slightly exceed the corresponding gas-phase equilibrium bond lengths (0.741 Å, 1.275 Å, and 1.988 Å, respectively), while remaining sufficiently restrictive to avoid counting non-bonded encounters as molecules.[S15]

Table S4: Molecular products identified during the production trajectory. Fractions are reported relative to the total number of identified H_2, HCl, and Cl_2 molecular events. Molecules were identified using the distance-based bonding cutoffs listed in the second column. HCl lifetime statistics are influenced by the periodic HCl-removal protocol and are therefore not reported.

Molecule	Cutoff (Å)	Count	Fraction (%)	Lifetime (ps)		
				Mean	Minimum	Maximum
H_2	0.90	13	0.5	1.93	0.01	24.99
HCl	1.30	1973	83.1	–	–	–
Cl_2	2.20	389	16.4	0.35	0.01	7.54
**Total**	–	**2375**	**100.0**	–	–	–

The statistics confirm that HCl is the dominant molecular product in the simulated decomposition pathway, accounting for 83.1% of all identified molecular events. Cl_2 formation is observed more frequently than H_2, but Cl_2 remains a secondary product with relatively low lifetime. The dominance of HCl supports the use of HCl removal as the principal volatile-loss channel in the MD protocol and is consistent with experimental studies identifying HCl as the dominant volatile species evolved during PVDC degradation. Note that because HCl is removed only at the end of each removal interval, some HCl molecules undergo further reactions before being removed; consequently, the formation event count exceeds the number of HCl molecules removed.

S2.2 Kinetic analysis of dehydrochlorination

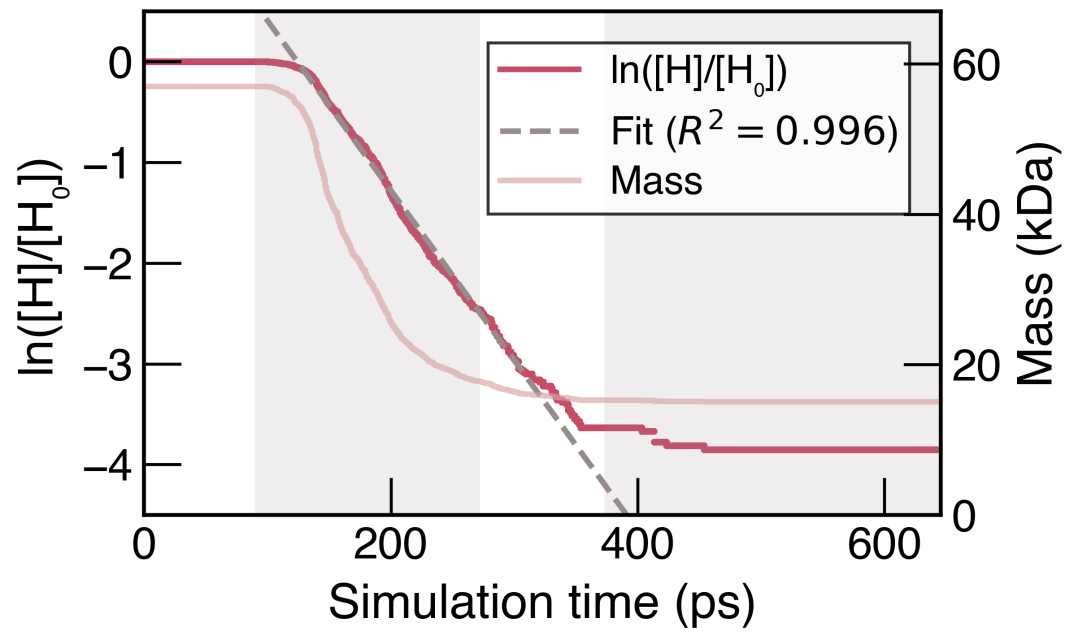

Figure S4: First-order kinetic analysis of simulated PVDC dehydrochlorination. Logarithmic decay of the hydrogen content in the simulation cell, used as a proxy for the remaining eliminable sites, is shown in burgundy. The gray dashed line shows a linear fit over 130 ps to 320 ps ($R^2 = 0.996$), and the translucent pink curve shows the cumulative mass change associated with HCl removal.

Since dehydrochlorination depletes hydrogen and chlorine stoichiometrically, the remaining hydrogen content provides a convenient proxy for the progress of dehydrochlorination. The main dehydrochlorination regime exhibits a nearly linear logarithmic decay of the reactant, supporting the use of an apparent pseudo-first-order description in the main text. The fitting window was selected to provide a linear regime, excluding both the initial induction period before sustained HCl evolution and the late-stage regime in which the remaining H/Cl-containing sites become depleted. Although the window was chosen empirically, it spans a substantial portion of the dehydrochlorination process. Nonetheless, this analysis should be interpreted as an empirical description of the simulated trajectory rather than as an experimental rate law, because the production protocol is accelerated and non-isothermal.

S2.3 Diffusivity analysis

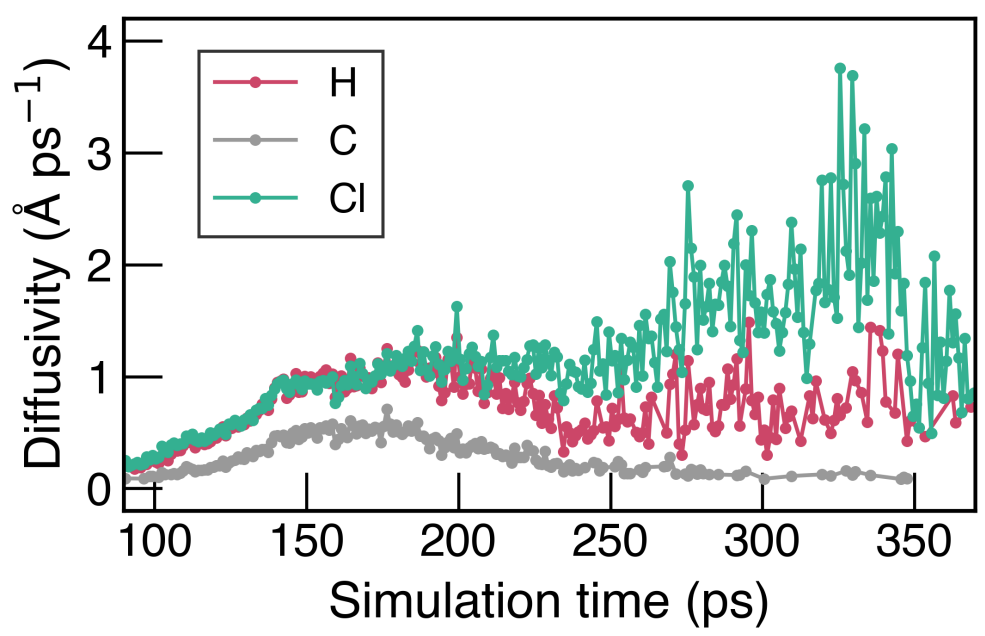

Figure S5: Segment-wise apparent mobility of carbon, hydrogen, and chlorine during PVDC pyrolysis. The apparent diffusivity of different elemental species during the NVT* stages was estimated from the slope of the mean-squared displacement (MSD) within each MD segment, using only segments with approximately linear MSD growth ($R^2 > 0.7$). Each point corresponds to the mid-point of an independent simulation segment. Since the MSD computation is reset at the start of each segment,[S16] the values should be interpreted as segment-wise apparent mobilities rather than long-time diffusion coefficients.

The segment-wise apparent mobility of carbon shows a modest rise during the period of active clustering, followed by a gradual decline as structural rigidity develops. Hydrogen remains more mobile than carbon but does not display the pronounced late-stage enhancement seen for chlorine. In contrast, the apparent diffusivity of chlorine is generally higher than that of hydrogen and much higher than that of carbon; although this metric includes both bonded and detached chlorine atoms, it is consistent with the idea that chlorine-containing species act as the primary mobile participants in HCl-forming events. After 250 ps, the chlorine diffusivity fluctuates strongly, with several high-mobility spikes. This behaviour suggests that late-stage dehydrochlorination is not uniform: some segments contain highly mobile chlorine species, while others exhibit more restricted chlorine mobility, consistent with a regime in which HCl formation becomes increasingly encounter-limited as reactive H-containing sites are depleted.

We note that the LAMMPS `compute msd` output reflects the mobility of all atoms in the specified group. Accordingly, the resulting curves should not be interpreted as direct comparisons between Cl· radicals and isolated hydrogen species. In particular, the high hydrogen mobility partly reflects the inherently larger thermal velocity of hydrogen and the presence of highly mobile gaseous HCl molecules that have formed but not yet been removed.

S2.4 Evolution of local coordination environments

To resolve how dehydrochlorination changes the immediate bonding environment of carbon atoms, we classified under-coordinated carbon sites according to their bonded neighbours.

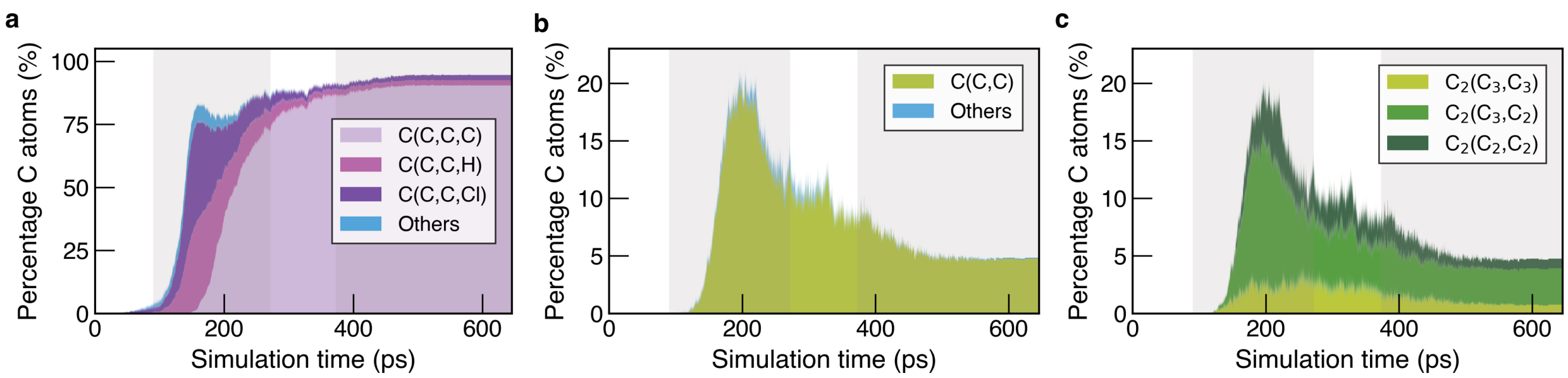

Figure S6: Temporal evolution of local coordination environments of carbon atoms. (**a**) Fractions of threefold-coordinated carbon atoms classified by neighbour environments. For example, C(C,C,Cl) denotes a threefold-coordinated carbon atom bonded to two carbon atoms and one chlorine atom. (**b**) Fractions of twofold-coordinated carbon atoms classified by neighbour environments. (**c**) Fractions of twofold-coordinated carbon atoms further classified by the coordination states of their carbon neighbours. For example, $C_2(C_3,C_2)$ denotes a twofold-coordinated carbon atom bonded to one threefold-coordinated carbon atom and one twofold-coordinated carbon atom. All percentages are normalized by the total number of carbon atoms in the simulation cell.

In Figure S6a, the very early stage of the reaction is dominated by C(C,C,Cl) environments, consistent with the initial loss of C–Cl bonds from fourfold-coordinated PVDC carbon atoms. As dehydrochlorination progresses, C(C,C,H) environments emerge, reflecting subsequent C–H bond cleavage. A minor population of other threefold-coordinated environments, primarily C(C,H,H) and C(C,Cl,Cl), is also observed; these species are attributed to transient C–C bond scission under high-temperature conditions.

At later stages, the growth of C(C,C,C) environments is accompanied by decreasing populations of both C(C,C,Cl) and C(C,C,H), indicating continued dehydrochlorination from already unsaturated threefold-coordinated carbon sites. This interpretation is supported by the concurrent increase in C(C,C) environments in Figure S6b. The subsequent decrease in C(C,C) environments, together with the growth of C(C,C,C) environments, suggests that these under-coordinated carbon sites are rapidly consumed through C–C cross-linking.

For twofold-coordinated carbon atoms, classification by immediate neighbours is less diagnostic because nearly all of them are bonded to two carbon atoms. Further classification according to the coordination states of neighbouring carbon atoms distinguishes $C_2(C_3,C_3)$ network bridges,

$C_2(C_3,C_2)$ chain–network junctions, and $C_2(C_2,C_2)$ chain-segment sites. The dominance of $C_2(C_3,C_2)$ environments indicates that twofold-coordinated carbon atoms occur more often at chain–network junctions than in extended chain segments; in other words, longer cumulene-like chains are disfavoured. The distinct behaviour of $C_2(C_3,C_3)$ sites is analysed further in Section S2.7, where their geometry, energetic signature, and lifetime are compared with other carbon local environments.

S2.5 Topological evolution

To establish the link between local topology and curvature in the developing carbon network, we analyse the evolution of ring populations alongside a local measure of non-planarity for threefold-coordinated carbon atoms using the out-of-plane (OOP) distance.

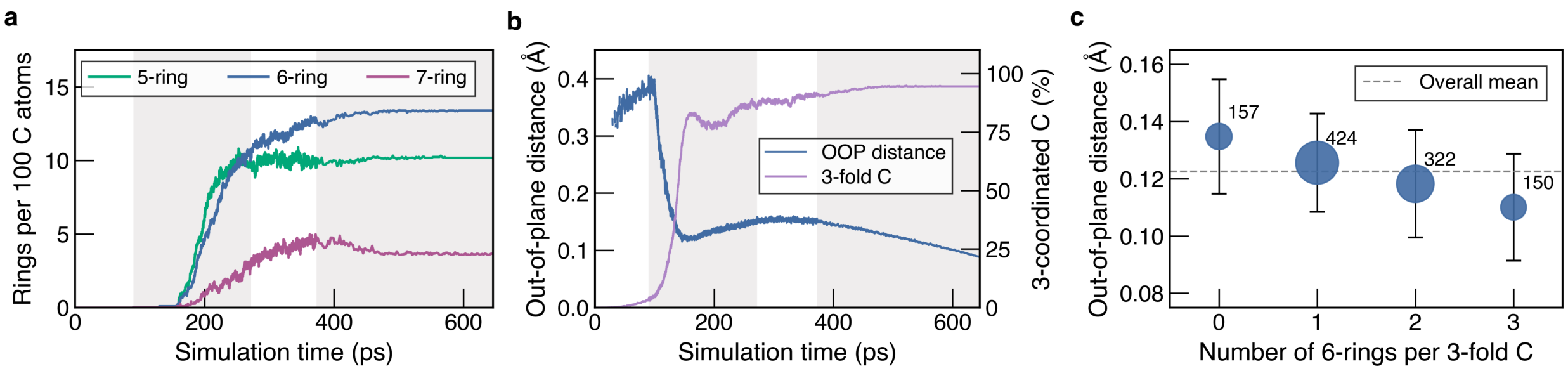

Figure S7: Topological analysis of the carbon network. (**a**) Evolution of the number of five-, six-, and seven-membered rings per 100 carbon atoms, computed using the ring-identification procedure described in Section S1.3. (**b**) Evolution of the average OOP distance of threefold-coordinated carbon atoms (*blue*). Evolution of the percentage of threefold-coordinated carbon atoms is shown in purple for reference. (**c**) Average OOP distance of threefold-coordinated carbon atoms during the final 170 ps of quenching (2000 K to 300 K), where the ring counts have stabilized. Carbon atoms are categorized by the number of six-membered rings to which they belong. Whiskers indicate standard deviations, labels indicate the average number of carbon atoms belonging to each category per frame, and are proportional to the marker size. The dashed line marks the average OOP distance across all threefold-coordinated carbon atoms in the same time window.

As shown in Figure S7a, carbon rings begin to form rapidly following the onset of carbon clustering. Five-membered rings appear slightly earlier and initially increase more rapidly in number than six-membered rings. In contrast, six-membered rings continue to accumulate over a longer period and ultimately become the most abundant ring motif in the final network. Seven-membered rings emerge more gradually and remain less abundant overall, but nevertheless constitute a persistent population of non-hexagonal defects throughout the later stages of pyrolysis. Interestingly, the slight decrease in the seven-membered-ring population at the start of quenching indicates that a fraction of non-hexagonal motifs remains structurally dynamic and can be annealed into more stable motifs through local rearrangement.

For each threefold-coordinated carbon atom, the OOP distance (shown in blue in Figure S7b) was defined as the perpendicular distance of the central atom from the plane formed by its three bonded carbon neighbours. Before sustained dehydrochlorination, transient C–Cl bond cleavage can generate a small amount of formally threefold-coordinated carbon atoms that still retain the local tetra-

hedral geometry of the precursor polymer, leading to relatively large OOP values. As dehydrochlorination progresses and threefold-coordinated carbon environments become increasingly prevalent, the average OOP distance decreases rapidly. A modest increase during the high-temperature stage is attributed to thermal distortion of the developing carbon network. During quenching, the OOP distance decreases continuously and stabilizes at approximately 0.1 Å, indicating partial relaxation toward locally planar sp^2-like environments under the constraints of the cross-linked network.

To assess the relationship between local ring topology and non-planarity, threefold-coordinated carbon atoms from the final 170 ps of the trajectory were grouped according to the number of six-membered rings in which they participate, and the corresponding OOP-distance distributions are shown in Figure S7c. A clear trend is observed: carbon atoms associated with a larger number of six-membered rings exhibit, on average, smaller OOP distances and therefore greater local planarity. This correlation supports the interpretation that incomplete hexagonal ordering contributes to local non-planarity and therefore curvature of the developing hard-carbon network.

S2.6 Atomic-energy descriptors of different local environments

To further characterize the relative stability of different carbon environments, we analysed the MACE-predicted per-atom energy contributions for carbon atoms with different local connectivity. Although atomic-energy decompositions in MLIPs are model-dependent and are not unique quantum-mechanical observables,[S17] they can provide useful comparative descriptors when interpreted within a fixed model. Here, we use these values to compare local carbon environments across the trajectory and to examine how local energetic trends correlate with ring topology and OOP distortion in the final carbon network.

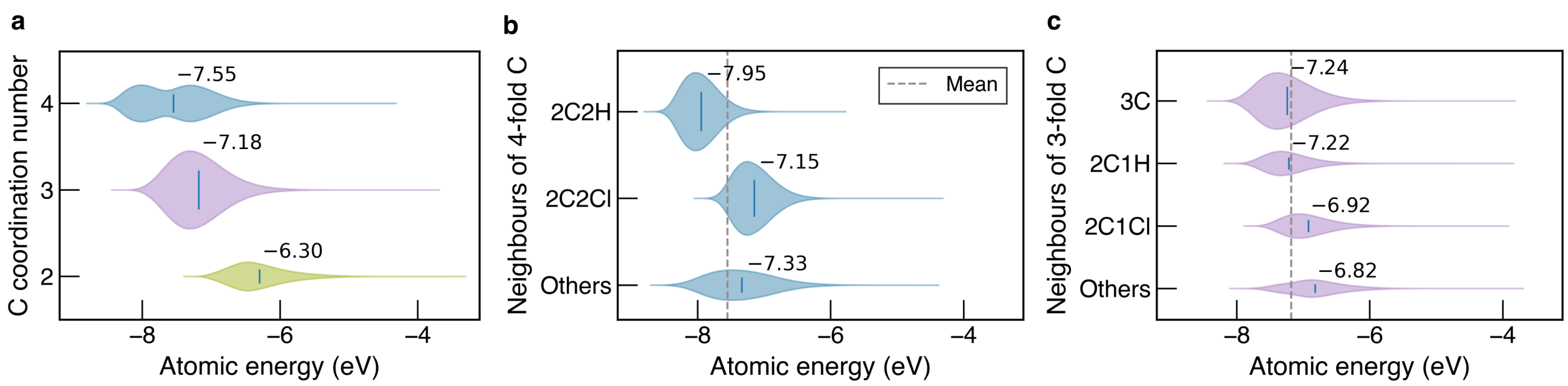

Figure S8: MACE-predicted atomic-energy distributions for carbon atoms in different local environments. (**a**) Atomic-energy distributions for carbon atoms categorized by coordination number across all simulation frames. Distributions are shown for fourfold-, threefold-, and twofold-coordinated carbon atoms; the blue line within each distribution indicates the corresponding mean value, and the distribution areas are proportional to the respective populations. (**b**) Atomic-energy distributions for fourfold-coordinated carbon atoms categorized by immediate neighbour environment: C(C,C,H,H), C(C,C,Cl,Cl), and other fourfold-coordinated environments. The gray dashed line indicates the mean atomic energy of all fourfold-coordinated carbon atoms. (**c**) Atomic-energy distributions for threefold-coordinated carbon atoms categorized as C(C,C,C), C(C,C,H), C(C,C,Cl), and other threefold-coordinated environments.

Fourfold-coordinated carbon atoms exhibit the lowest average MACE atomic-energy contributions among the coordination states considered. This likely reflects their intrinsic stability as well as their predominance in the intact PVDC precursor during the low-temperature stage of the simulation. Within this class (Figure S8b), C(C,C,Cl,Cl) environments are systematically higher in energy than C(C,C,H,H) environments, consistent with the greater susceptibility of C–Cl bonds to activation and cleavage during dehydrochlorination. The remaining fourfold-coordinated environments show broader and generally higher-energy distributions, as they were primarily generated through radical recombination during early-stage cross-linking processes.

Although threefold-coordinated carbon atoms exhibit higher average atomic-energy contributions

than fourfold-coordinated carbon atoms when sampled over the full trajectory, this comparison should be interpreted with care as threefold-coordinated environments are populated during the high-temperature dehydrochlorination and carbonization stages. Within the threefold-coordinated class (Figure S8c), C(C,C,C) environments are the lowest in energy, followed by C(C,C,H) and C(C,C,Cl), consistent with the stabilization of carbon atoms as they become incorporated into the carbon network. Twofold-coordinated carbon atoms are not further classified by immediate neighbour identity, because the vast majority are bonded to two carbon atoms; their secondary-neighbour environments are analyzed separately in Section S2.7.

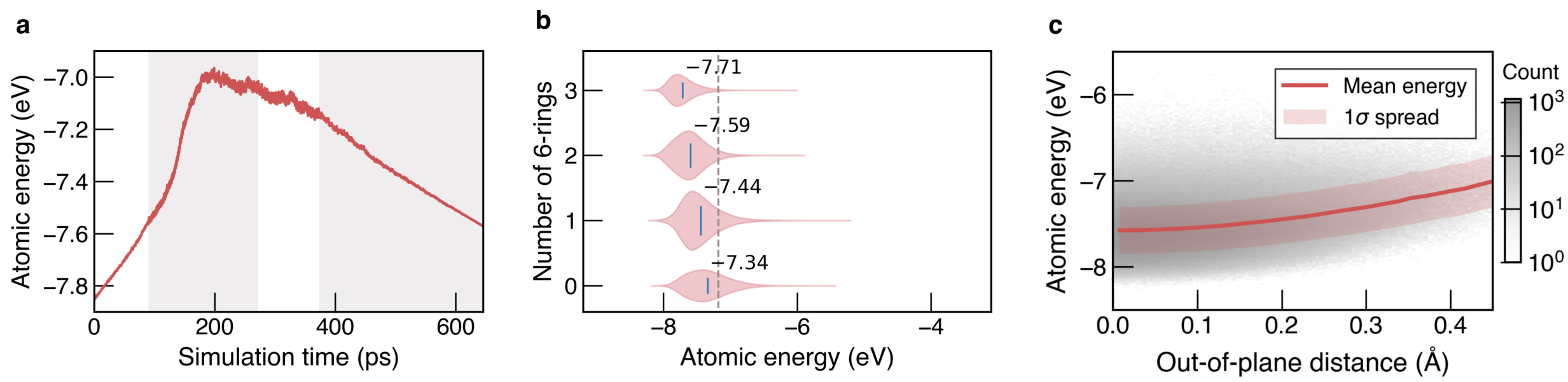

Figure S9: Temporal evolution of MACE atomic energies and their correlation with local topology. (**a**) Evolution of the frame-averaged MACE atomic-energy contributions of carbon atoms. (**b**) MACE atomic-energy distributions of threefold-coordinated carbon atoms from the final 170 ps of the trajectory, categorized by the number of six-membered rings in which each atom participates. (**c**) Correlation between MACE atomic-energy contribution and OOP distance for threefold-coordinated carbon atoms from the final 170 ps. The heat map shows the occurrence of individual carbon environments, with denser colours reflecting higher abundance. The pink line shows the mean atomic energy as a function of OOP distance; the shaded region denotes one standard deviation.

The mean carbon atomic energy initially increases with temperature, reflecting thermal bond activation in the precursor together with the progressive conversion of fourfold-coordinated carbon atoms into threefold-coordinated environments. The emergence of twofold-coordinated carbon atoms (around 160 ps; Figure 3 of the main manuscript) coincides with a second sharp increase in the average atomic energy, consistent with their role as energetically unfavourable intermediates. At later stages, the average energy decreases steadily as twofold-coordinated sites are consumed through C–C cross-linking and the carbon network rearranges toward more stable threefold-coordinated configurations.

Figures S9b,c show a clear correlation between ring topology, non-planarity, and MACE atomic-energy contributions during the 2000 K to 300 K quenching stage, where the ring populations

have largely stabilized. Threefold-coordinated carbon atoms participating in a larger number of six-membered rings exhibit both lower average atomic-energy contributions and lower maximum atomic energies. Similarly, the increasingly positive slope of the mean-energy curve in Figures S9c suggests that threefold-coordinated carbon atoms with larger OOP distances are associated with increasingly higher-energy local environments. Together, these trends indicate that hexagon-rich, locally planar motifs are energetically more favourable within the MACE energy decomposition, whereas incompletely hexagonal topologies are associated with enhanced curvature and higher local energetic cost.

S2.7 Structural signatures of under-coordinated carbon intermediates

To further characterize the local structures during pyrolysis, we analysed the evolution of C–C bond lengths, C-centred bond angles, and MACE-predicted atomic-energy contributions for carbon atoms with different coordination numbers. As twofold-coordinated carbon atoms show the largest deviations from idealized local geometries, we further resolved these environments according to the coordination states of their neighbouring carbon atoms, as defined in Section S2.4.

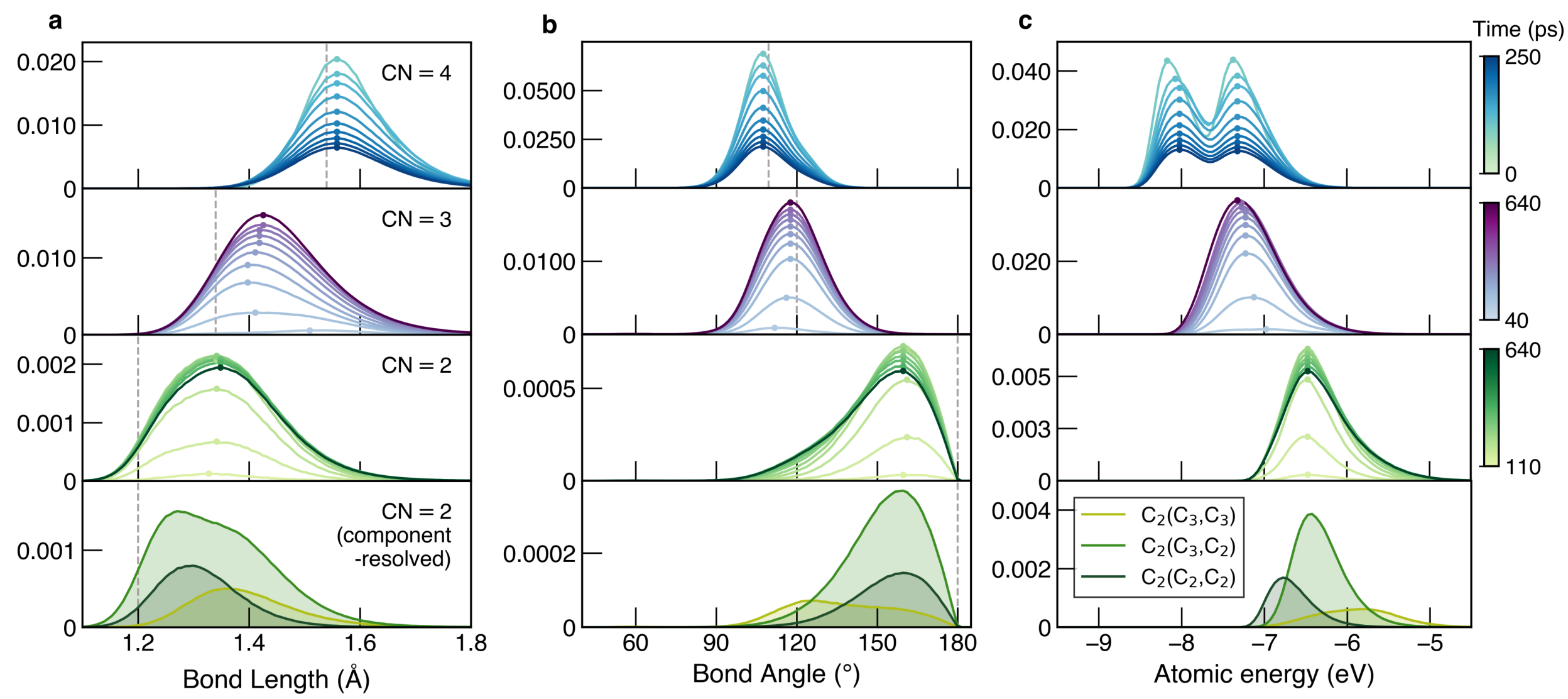

Figure S10: Geometric and energetic signatures of local carbon environments during PVDC pyrolysis. Time-resolved cumulative distributions of local descriptors: (**a**) C–C bond lengths, (**b**) C-centred bond angles, and (**c**) MACE-predicted atomic-energy contributions. In each column, the upper three panels show distributions grouped by carbon coordination number. Curves correspond to cumulative kernel-density estimates, normalized by the number of contributing atoms accumulated up to each time interval, and are coloured according to simulation time. Different time windows, indicated by the corresponding colour bars on the right, are used for different coordination states to focus on the periods in which those environments are appreciably populated: 0 ps to 250 ps for fourfold-coordinated carbon, 40 ps to 640 ps for twofold-coordinated carbon, and 110 ps to 640 ps for threefold-coordinated carbon. Scatter points mark the evolving maxima of the corresponding distributions. The lower panels resolve the same descriptors for twofold-coordinated carbon atoms according to secondary-neighbour connectivity, without time resolution for better visual clarity. Gray dashed lines in panels (**a**) and (**b**) indicate reference values for the $C_{sp^3}–C_{sp^3}$ (ethane), $C_{sp^2}–C_{sp^2}$ (ethylene), and $C_{sp}–C_{sp}$ (acetylene) bond lengths;[S18] and the tetrahedral, trigonal-planar, and linear C-centred bond angles, respectively.

In Figure S10a, the C–C bond lengths between fourfold-coordinated carbon atoms remain close to typical $C_{sp^3}–C_{sp^3}$ bond lengths during the early stage of the simulation, while their population decreases as dehydrochlorination proceeds. Threefold-coordinated carbon atoms become increasingly populated during carbonization, and their characteristic C–C bond length approaches ap-

proximately 1.42 Å toward the end of the trajectory, close to $C_{sp^2}-C_{sp^2}$ bond lengths observed in graphite. The small shift to longer bond lengths for both CN = 4 and CN = 3 may reflect residual tensile strain or the relatively open hard-carbon network formed under the density-calibrated protocol. The bond-length distribution of twofold-coordinated carbon remains broader despite lower population, and noticeably shifted relative to the idealized sp-bonded reference, indicating that these sites are not simply relaxed linear sp-carbon motifs, but strained intermediates embedded within the carbon network.

The corresponding bond-angle distributions in Figure S10b support this interpretation. Fourfold-coordinated carbon atoms remain close to the tetrahedral reference angle, with minimal variation with simulation time. At early times, some threefold-coordinated carbon atoms retain bond angles closer to tetrahedral geometry, consistent with local configurations that have lost a substituent but have not yet fully relaxed toward trigonal-planar geometry (Figure S7b). As the network matures, the threefold-coordinated population shifts toward angles slightly below 120°, consistent with locally sp^2-like carbon environments distorted by curvature and topological disorder. In contrast, twofold-coordinated carbon atoms show the largest deviation from ideal linear geometry, indicating that these sites are strongly bent and constrained by the surrounding network.

The MACE atomic-energy distributions in Figure S10c provide a complementary model-based descriptor of local stability. Fourfold-coordinated carbon atoms show distinct bimodal populations, consistent with the different energetic environments of C(C,C,H,H) and C(C,C,Cl,Cl) motifs discussed in Figure S8. As the temperature increases, both populations shift to higher energy, reflecting thermal bond activation. Threefold-coordinated carbon atoms initially sample relatively high-energy environments, because early threefold-coordinated species are dominated by C(C,C,Cl)-type motifs generated by C–Cl cleavage. At later times, the distribution shifts to lower energies as more stable C(C,C,C) environments form and the network relaxes during quenching.

The anomalous geometry and high energy of twofold-coordinated carbon atoms motivated a more detailed classification by secondary-neighbour connectivity in the last panel of each column in Figure S10. Among these environments, $C_2(C_2,C_2)$ sites show the shortest C–C bonds, consistent with greater cumulene-like s-character in the bonding. $C_2(C_3,C_2)$ environments display a broader, bimodal bond-length distribution, reflecting the asymmetry between the bond to a twofold-

coordinated neighbour and the bond to a threefold-coordinated network site. $C_2(C_3,C_3)$ environments show the longest C–C bonds. Moreover, in contrast to the other two C_2 environments, whose bond-angle distributions peak near 160°, the bond-angle distribution of $C_2(C_3,C_3)$ sites is centred much closer to trigonal values. This suggests that these $C_2(C_3,C_3)$ atoms are geometrically more similar to distorted threefold-coordinated network sites, but lack one formal C–C connection under the adopted bonding criterion.

This interpretation is reinforced by the atomic-energy analysis. $C_2(C_3,C_3)$ sites have the highest MACE atomic-energy contributions among the twofold-coordinated environments and are substantially higher in energy than typical threefold-coordinated carbon atoms.

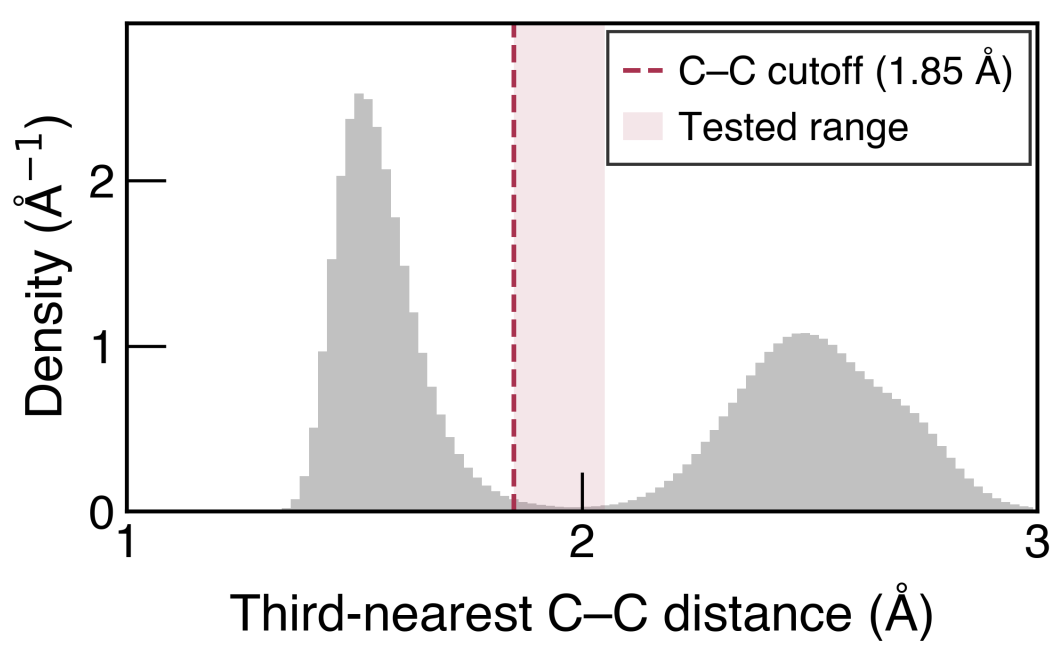

C–C cutoff (Å)	Retained (%)	Median ΔE (eV)
1.85	100.0	1.27
1.90	85.9	1.33
2.00	73.1	1.37
2.05	69.1	1.37

Classification	Fraction (%)	Median ΔE (eV)
Cutoff-stable	69.1	1.40
Cutoff-sensitive	30.9	0.94

Figure S11: Sensitivity of $C_2(C_3,C_3)$ environments to the C–C cutoff. *Left:* Distribution of third-nearest C–C distances throughout the simulation. The dashed line indicates the cutoff used for the coordination analysis. *Top right:* Fraction of $C_2(C_3,C_3)$ sites identified at 1.85 Å that retain this classification as the cutoff is increased. For each site, ΔE is its atomic energy relative to the mean atomic energy of C_3 atoms in the same frame; median values are reported over all sites. *Bottom right:* Comparison of cutoff-stable and cutoff-sensitive $C_2(C_3,C_3)$ sites at 2.05 Å.

To assess the sensitivity of the classification of $C_2(C_3,C_3)$ sites to the fixed C–C cutoff, we first examined the distribution of third-nearest C–C distances throughout the trajectory (Figure S11 left). The distribution shows a well-defined first-neighbour peak separated from the next coordination shell by a deep minimum containing the adopted cutoff of 1.85 Å, although a finite population remains within this region under the high-temperature conditions sampled. We therefore tested $C_2(C_3,C_3)$ sites specifically by increasing the cutoff to 2.05 Å (Figure S11 right). The fraction of retained sites decreases progressively with increasing cutoff, while their median ΔE increases from 1.27 eV to 1.37 eV. This behaviour is consistent with the third neighbours of $C_2(C_3,C_3)$ environments spanning a continuous range of separations within the inter-shell minimum. Importantly, even the 30.9% of sites that change classification at 2.05 Å remain substantially higher in energy

than threefold-coordinated carbon in the same frame, with a median ΔE of 0.94 eV. Thus, varying the cutoff changes whether some elongated C–C contacts are classified as bonded, but not their high-energy character. The identified $C_2(C_3,C_3)$ sites therefore represent transient configurations sampling continuously varying C–C separations during bond rearrangement.

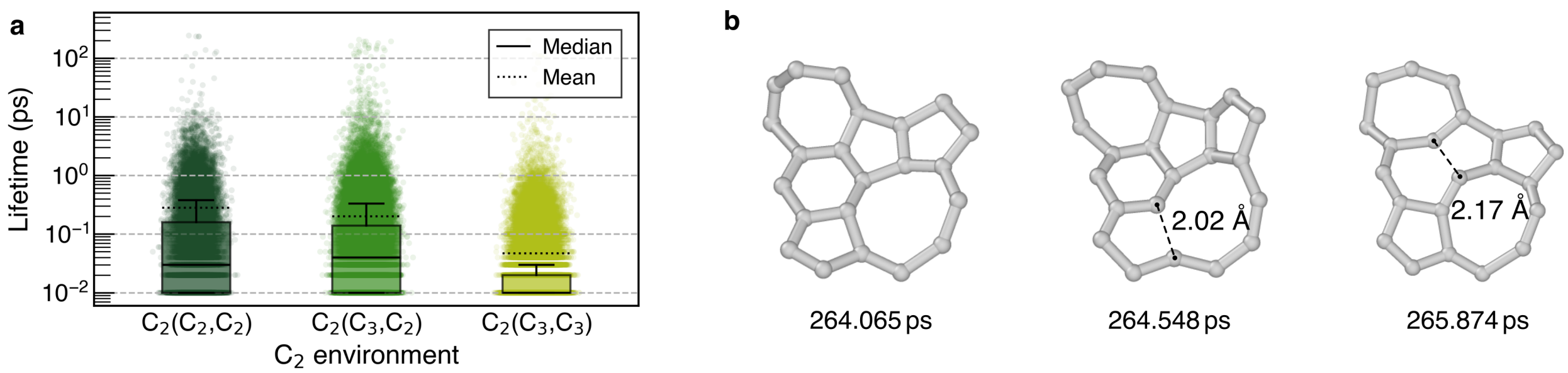

Figure S12: Lifetimes of under-coordinated carbon intermediates (**a**) Lifetime distributions of twofold-coordinated carbon atoms classified by secondary-neighbour connectivity. Boxes span the interquartile range; solid and dotted lines denote the median and mean, respectively; whiskers extend to 1.5× the interquartile range. (**b**) Representative fragments illustrating the transient appearance and disappearance of $C_2(C_3,C_3)$ environments during high-temperature network rearrangement.

Representative fragments in Figure S12b show that transient C–C bond breaking within an already developed carbon network, for example near a larger ring motif, can produce $C_2(C_3,C_3)$ sites with a neighbouring C–C separation well beyond the bonding cutoff of 1.85 Å. The lifetime analysis in Figure S12a further shows that $C_2(C_3,C_3)$ environments have much shorter mean, median, and maximum lifetimes than the other C_2 environments. Thus, $C_2(C_3,C_3)$ species are best interpreted as transient high-energy intermediates associated with local bond rearrangement.

S3 Supplementary references

S1. Stenczel, T. K.; El-Machachi, Z.; Liepuoniute, G.; Morrow, J. D.; Bartók, A. P.; Probert, M. I. J.; Csányi, G.; Deringer, V. L. Machine-Learned Acceleration for Molecular Dynamics in CASTEP. *J. Chem. Phys.* **2023**, *159*, 044803.

S2. Rowe, P.; Deringer, V. L.; Gasparotto, P.; Csányi, G.; Michaelides, A. An Accurate and Transferable Machine Learning Potential for Carbon. *J. Chem. Phys.* **2020**, *153*, 034702.

S3. Ibragimova, R.; Kuklin, M. S.; Zarrouk, T.; Caro, M. A. Unifying the Description of Hydrocarbons and Hydrogenated Carbon Materials with a Chemically Reactive Machine Learning Interatomic Potential. *Chem. Mater.* **2025**, *37*, 1094–1110.

S4. Kim, S.; Chen, J.; Cheng, T.; Gindulyte, A.; He, J.; He, S.; Li, Q.; Shoemaker, B. A.; Thiessen, P. A.; Yu, B.; Zaslavsky, L.; Zhang, J.; Bolton, E. E. PubChem 2025 update. *Nucleic Acids Res.* **2025**, *53*, D1516–D1525.

S5. Williams, N. J.; Kabalan, L.; Stojanovic, L.; Zólyomi, V.; Pyzer-Knapp, E. O. Hessian QM9: A Quantum Chemistry Database of Molecular Hessians in Implicit Solvents. *Sci. Data* **2025**, *12*, 9.

S6. Batatia, I.; Kovacs, D. P.; Simm, G.; Ortner, C.; Csanyi, G. MACE: Higher Order Equivariant Message Passing Neural Networks for Fast and Accurate Force Fields. *Adv. Neural. Inf. Process. Syst.* **2022**, *35*, 11423–11436.

S7. Broyden, C. G. The Convergence of a Class of Double-rank Minimization Algorithms 1. General Considerations. *IMA J. Appl. Math.* **1970**, *6*, 76–90.

S8. Fletcher, R. A New Approach to Variable Metric Algorithms. *Comput. J.* **1970**, *13*, 317–322.

S9. Goldfarb, D. A Family of Variable-Metric Methods Derived by Variational Means. *Math. Comput.* **1970**, *24*, 23–26.

S10. Shanno, D. F. Conditioning of Quasi-Newton Methods for Function Minimization. *Math. Comput.* **1970**, *24*, 647–656.

S11. Franzblau, D. S. Computation of ring statistics for network models of solids. *Phys. Rev. B* **1991**, *44*, 4925–4930.

S12. Trucano, P.; Chen, R. Structure of Graphite by Neutron Diffraction. *Nature* **1975**, *258*, 136–137. DOI: 10.1038/258136a0.

S13. Putman, K. J.; Sofianos, M. V.; Rowles, M. R.; Harris, P. J. F.; Buckley, C. E.; Marks, N. A.; Suarez-Martinez, I. Pulsed Thermal Treatment of Carbon up to 3000 °C Using an Atomic Absorption Spectrometer. *Carbon* **2018**, *135*, 157–163. DOI: 10.1016/j.carbon.2018.03.060.

S14. Franklin, R. E. Crystallite Growth in Graphitizing and Non-Graphitizing Carbons. *Proc. R. Soc. London, Ser. A* **1951**, *209*, 196–218. DOI: 10.1098/rspa.1951.0197.

S15. Huber, K. P.; Herzberg, G. *Molecular Spectra and Molecular Structure IV. Constants of Diatomic Molecules*; Van Nostrand Reinhold: New York, 1979.

S16. Thompson, A. P.; Aktulga, H. M.; Berger, R.; Bolintineanu, D. S.; Brown, W. M.; Crozier, P. S.; in 't Veld, P. J.; Kohlmeyer, A.; Moore, S. G.; Nguyen, T. D.; Shan, R.; Stevens, M. J.; Tranchida, J.; Trott, C.; Plimpton, S. J. LAMMPS - a flexible simulation tool for particle-based materials modeling at the atomic, meso, and continuum scales. *Comput. Phys. Commun.* **2022**, *271*, 108171.

S17. Chong, S.; Grasselli, F.; Ben Mahmoud, C.; Morrow, J. D.; Deringer, V. L.; Ceriotti, M. Robustness of Local Predictions in Atomistic Machine Learning Models. *J. Chem. Theory Comput.* **2023**, *19*, 8020–8031.

S18. Allen, F. H.; Kennard, O.; Watson, D. G.; Brammer, L.; Orpen, A. G.; Taylor, R. Tables of bond lengths determined by X-ray and neutron diffraction. Part 1. Bond lengths in organic compounds. *J. Chem. Soc., Perkin Trans. 2* **1987**, S1–S19.
